# Prognostic analysis of sepsis-induced myocardial injury patients using propensity score matching and doubly robust analysis with machine learning-based risk prediction model development

**DOI:** 10.3389/fmed.2025.1555103

**Published:** 2025-02-19

**Authors:** Pan Guo, Li Xue, Fang Tao, Kuan Yang, YuXia Gao, Chongzhe Pei

**Affiliations:** ^1^Graduate School, Tianjin Medical University, Tianjin, China; ^2^Department of Cardiology, Qinhuangdao First Hospital, Qinhuangdao, China; ^3^Department of Cardiology, Tianjin Medical University General Hospital, Tianjin, China; ^4^Medical Department, Qinhuangdao First Hospital, Qinhuangdao, China

**Keywords:** sepsis, myocardial injury, prognosis, propensity score matching, doubly robust analysis, machine learning

## Abstract

**Background:**

Sepsis-induced myocardial injury (SIMI) is a severe and common complication of sepsis; However, its definition remains unclear. Prognostic analyses may vary depending on the definition applied. Early prediction of SIMI is crucial for timely intervention, ultimately improving patient outcomes. This study aimed to evaluate the prognostic impact of SIMI and develop validated predictive models using advanced machine learning (ML) algorithms for identifying SIMI in critically ill sepsis patients.

**Methods:**

Data were sourced from the Medical Information Mart for Intensive Care IV (MIMIC-IV, v3.0) database. Patients meeting Sepsis-3.0 criteria were included, and SIMI was defined as troponin T (TNT) levels ≥0.1 ng/mL. Prognostic evaluation involved propensity score matching, inverse probability weighting, doubly robust analysis, logistic regression, and Cox regression. Patients were divided into training and testing datasets in a 7:3 ratio. Least absolute shrinkage and selection operator (LASSO) regression was used for variable selection to simplify the model. Twelve hyperparameter-tuned ML models were developed and evaluated using visualized heatmaps. The best-performing model was deployed as a web-based application.

**Results:**

Among 2,435 patients analyzed, 571 (23.45%) developed SIMI following intensive care unit (ICU) admission. Boruta and LASSO identified 46 and 10 key variables, respectively, for prognostic and predictive modeling. Doubly robust analysis revealed significantly worse short- and intermediate-term outcomes for SIMI patients, including increased in-ICU mortality [odds ratio (OR) 1.39, 95% confidence interval (CI) 1.02–1.85, *p* < 0.05], 28-day mortality (OR 1.35, 95% CI 1.02–1.79, *p* < 0.05), and 180-day mortality [hazard ratio (HR) 1.21, 95% CI 1.01–1.44, *p* < 0.05]. However, one-year mortality showed no significant difference (HR 1.03, 95% CI 0.99–1.08, *p* = 0.169). The XGBoost model outperformed others, achieving an area under the receiver operating characteristic curve (AUROC) of 0.83 (95% CI 0.79–0.87). SHapley Additive exPlanations (SHAP) analysis highlighted the top five predictive features: creatine kinase-myocardial band (CKMB), creatinine, alanine aminotransferase (ALT), lactate, and blood urea nitrogen (BUN). A web-based application was subsequently developed for real-world use.

**Conclusion:**

SIMI significantly worsens patient prognosis, while the XGBoost model demonstrated excellent predictive performance. The development of a web-based application provides clinicians with a practical tool for timely intervention, potentially improving outcomes for septic patients.

## Introduction

Sepsis is one of the most common diseases and remains the leading cause of death in intensive care unit (ICU) patients, resulting in a significant global health burden (1). Patients with sepsis present with multiple life-threatening organ dysfunctions due to a dysregulated response to infection (2). Sepsis is a critical condition that often leads to severe cardiovascular complications, including cardiac injury. This injury is not merely a transient phenomenon; rather, it has far-reaching implications for both early-term and long-term prognoses. Studies have shown that patients with sepsis frequently exhibit signs of cardiac dysfunction that can be detected using various biomarkers and clinical assessments. For instance, elevated troponin levels, which are indicative of myocardial injury, have been associated with increased mortality rates in patients with sepsis. A cohort study demonstrated that undiagnosed myocardial infarction in critically ill patients with cardiovascular disease was significantly correlated with lower long-term survival rates (3). Troponin testing is a crucial tool for detecting sepsis-induced myocardial dysfunction in patients with septic shock (4). Thus, early recognition of and aggressive intervention for sepsis-induced myocardial injury (SIMI) are of paramount importance.

In the ICU, echocardiography requires high-quality imaging and precise operational skills. Operator-dependent errors in echocardiographic assessments are inevitable and may lead to misinterpretation of a patient’s condition. For example, the quality of cardiac ultrasound images may be compromised in patients with cardiogenic shock, potentially affecting the evaluation of left ventricular function (5, 6). When evaluating sepsis-associated cardiomyopathy, utilization of ICU scoring systems is critical. While the Acute Physiology and Chronic Health Evaluation II (APACHE II) and Sequential Organ Failure Assessment (SOFA) scoring systems demonstrate robust efficacy in evaluating disease severity and prognosticating outcomes in patients with sepsis (7), they lack specificity for SIMI.

In contrast to the numerous etiologies of cardiomyopathy, SIMI exhibits unique reversibility. This distinctive feature underscores the critical importance of early detection and prompt intervention in patients with septic shock who develop myocardial dysfunction (8). Given the current absence a definitive definition of SIMI and effective predictive models (9), we approached this research from a clinical perspective, utilizing troponin as a diagnostic marker to explore the prognosis of patients with SIMI and to develop an early identification model for SIMI.

## Methods

### Data source

This retrospective observational study utilized data from the Medical Information Mart for Intensive Care IV (MIMIC-IV, version 3.0) database, an updated version of MIMIC-III. This database encompasses comprehensive critical care data for ICU patients at the Beth Israel Deaconess Medical Center (BIDMC) from 2008 to 2022. It contains detailed patient records including laboratory measurements, administered medications, vital signs, and additional clinical parameters. Access to the database was granted to author PG following the completion of the requisite data usage agreement and Collaborative Institutional Training Initiative (CITI) certification. Given the de-identified nature of all patient data, informed consent was not required (10).

### Study population

#### Inclusion criteria

Patients with sepsis meeting the Sepsis-3.0 criteria (11).

#### Exclusion criteria

Non-first hospitalization; absence of ICU records; ICU stay time less than 1 day; diseases resulting in elevated troponin T levels [including myocarditis, pericarditis, coronary disease, congestive heart failure, rhabdomyolysis, end-stage chronic kidney disease (CKD), burn, stroke, pulmonary embolism, cancer, myocardial contusion, fibrillation, takotsubo syndrome, infiltrative cardiomyopathies, aortic dissection, cardioversion, cardiothoracic surgery]; absence of troponin T (TNT) examination.

#### Diagnostic criteria

**Figure 1:**
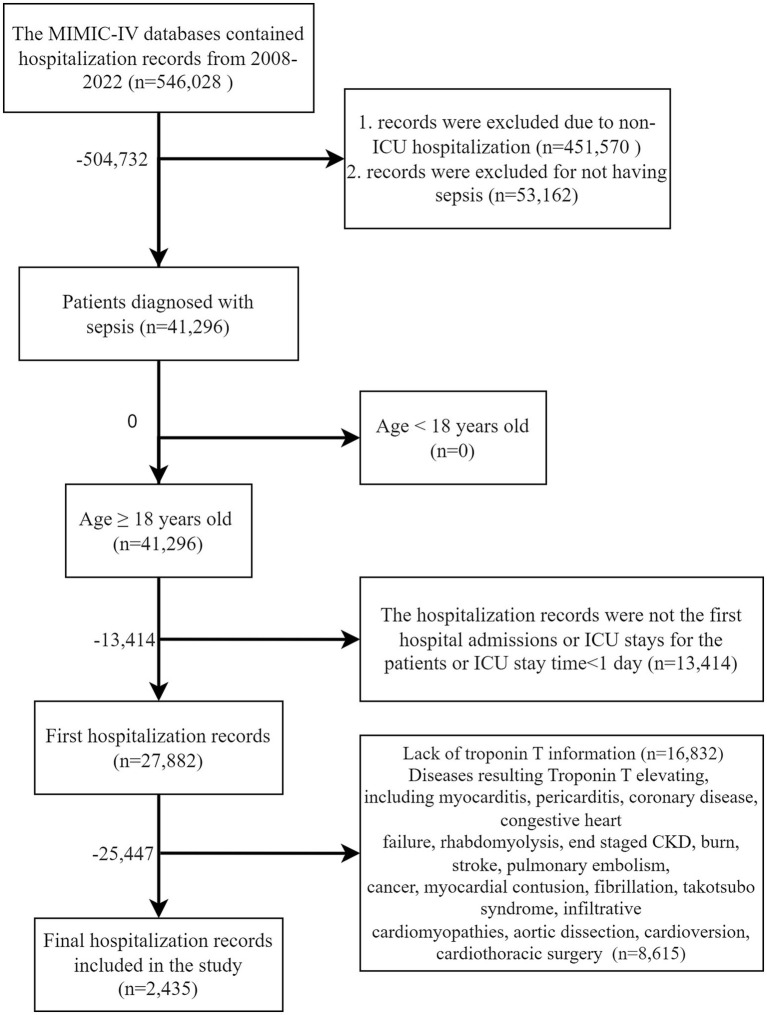
Flow chart of study design.

Currently, there is no established or widely accepted definition for SIMI. In clinical settings, TNT serves as a marker for assessing myocardial damage in patients with sepsis. Research has demonstrated a correlation between elevated TNT levels and increased mortality in these patients (4). According to the MIMIC-IV database, a TNT level of ≥0.1 ng/mL indicates myocardial injury, and thus we use this threshold as the diagnostic criterion for SIMI. The details of population selection are shown in Figure 1.

### Data extraction

Data extraction was conducted using PostgreSQL 14 with SQL queries (Berkeley, California, United States). The resulting dataset comprised patient demographics, ICU length of stay, complications, laboratory test results, treatments, and other pertinent clinical information. For the prognostic analysis of patients with SIMI, the maximum and minimum values of laboratory results were retrieved to evaluate their impact on patient outcomes. In constructing the machine learning (ML) models, only the initial laboratory results obtained at ICU admission were utilized because these early values are promptly accessible and enable timely assessment through clinical prediction models.

### Statistical analysis

The Kolmogorov–Smirnov test was employed to assess the normality of continuous variables. Data with normal distribution were reported as mean ± standard deviation, while non-normally distributed variables were expressed as median and interquartile range [IQR, M (P25, P75)]. Levene’s test was performed to evaluate the homogeneity of variances. For comparisons between two groups, Student’s *t*-test was applied to continuous variables meeting the criteria of normality and homogeneity of variances; otherwise, the Mann–Whitney *U* test was used. Categorical variables were summarized as frequencies and percentages, with Fisher’s exact test applied for sample sizes under 40 and the chi-square test for larger samples. Missing data were handled using multiple imputation with the “mice” package in R, excluding variables with over 40% missingness from imputation and modeling. A total of 100 imputations were conducted to ensure robust estimates.

The gradient boosted model (GBM) was utilized to calculate propensity scores for SIMI. To identify key variables significantly associated with the outcome, the Boruta feature selection method was applied using the Boruta package in R. This method ensures the inclusion of statistically relevant features while reducing noise and redundancy from excessive variables. An inverse probability of treatment weighting (IPTW) approach was used to create a weighted cohort based on propensity scores. Covariate imbalances between the original and weighted cohorts were assessed using standardized mean differences (SMDs) to evaluate the effectiveness of propensity score adjustment. Logistic regression or Cox regression was subsequently performed on the weighted cohort, adjusting for any residual imbalances. This doubly robust analysis was carried out using the “survey” package for weighting and logistic regression through the “stats” package. The doubly robust estimator ensures unbiased effect estimates even when only one of the underlying models is correctly specified (12). Cox proportional hazards models were constructed using the “survival” package, which also facilitated testing the proportional hazards (PH) assumption via functions such as “cox.zph.” In cases where time-dependent covariates violated the PH assumption, appropriate methods such as time-dependent effects or stratification were employed to enhance model fit and ensure accurate hazard ratio (HR) estimation.

### Machine learning model construction

The dataset was split into a 70% training set and a 30% test set. To address the impact of high-dimensional data on ML algorithm performance, the least absolute shrinkage and selection operator (LASSO) method was applied for variable selection prior to model construction. This technique minimizes prediction error while applying constraints that shrink some coefficients to zero, effectively selecting only variables with non-zero coefficients as robust predictors. The LASSO analysis, performed in the R environment, utilized a 10-fold cross-validation process with centered and scaled variables to determine the optimal tuning parameter lambda. The implementation of LASSO regression was facilitated by the “glmnet” package, which simultaneously enables shrinkage and variable selection.

Following variable selection, the Synthetic Minority Oversampling Technique (SMOTE) was applied as a preprocessing step to balance the sample distribution between the SIMI and non-SIMI populations within the training dataset. We employed 12 ML methods for model construction based on the training set: decision tree (DT), random forest (RF), extreme gradient boosting (XGBoost), light gradient boosting machine (LightGBM), support vector machines (SVM), multilayer perceptron (MLP), K-nearest neighbors (KNN), logistic regression, LASSO regression, ridge regression, elastic net (ENet), and a stacking ensemble (which integrates KNN, RF, and logistic regression). Each ML model underwent grid search for hyperparameter tuning and quintuple cross-validation to optimize performance and ensure reliability. The area under the receiver operating characteristic curve (AUROC) was employed to assess the models’ predictive accuracy during validation. AUROC values range from 0.5 to 1.0, with higher values indicating better predictive capability. The SHAP (SHapley Additive exPlanations) algorithm was applied to interpret the final model with the highest efficacy, assigning each variable a corresponding SHAP value to quantify its influence on prediction accuracy. A SHAP summary plot was created to illustrate the contribution of each feature to the model’s performance. All ML analyses were conducted within the framework of the “tidymodels” package in R, with SHAP analysis performed using Python. The final model’s application development and web deployment^1^ were conducted using the “shiny” and “DT” packages in R, making this application accessible to medical centers worldwide for model implementation.

Statistical analyses in this study were conducted using R software (version 4.4.1; R Foundation for Statistical Computing, Vienna, Austria) and Python (version 3.12.3; Python Software Foundation, Wilmington, DE, United States). A two-tailed test was utilized, with a *p*-value of less than 0.05 deemed statistically significant.

## Results

### Baseline characteristics

After rigorous screening, a total of 2,435 patients were ultimately included in the final analysis, as detailed in Figure 1. Of the patients enrolled in our study, 571 (23.45%) developed SIMI following ICU admission, as illustrated in Table 1; Supplementary Tables S1–S3. The initial measurements of each parameter enumerated in Table 1 were employed as variables for the ML model, since the utilization of these initial measurements facilitates the early prediction of SIMI. Additionally, we employed the maximum and minimum values of various measurements taken during hospitalization as covariates [with the exception of TNT, creatinine, and C-reactive protein (CRP), for which only the maximum values were included, since the minimum values hold little significance for patient prognosis] to assess the outcomes of patients with SIMI. This approach allows for a more comprehensive capture of the patients’ status throughout their hospital stay, thereby enhancing the accuracy of the prognostic analysis.

**Table 1:** Baseline characteristics before and after propensity score matching of two cohorts.

	Before matching			After matching		
	Non-SIMI (*N* = 1,864)	SIMI (*N* = 571)	SMD	Non-SIMI (*N* = 408)	SIMI (*N* = 408)	SMD
Age	61.00 [50.00, 74.00]	63.00 [51.00, 75.00]	0.049	62.00 [52.00, 74.00]	64.00 [51.00, 76.00]	0.012
Gender (female)	**847 (45.44%)**	**232 (40.63%)**	**0.097**	163 (42.34%)	163 (42.34%)	<0.001
Mechanical ventilation (yes)	**1,024 (54.94%)**	**415 (72.68%)**	**0.376**	263 (68.31%)	245 (63.64%)	0.099
Continuous renal replacement therapy (yes)	**69 (3.70%)**	**50 (8.76%)**	**0.21**	**43 (11.17%)**	**23 (5.97%)**	**0.186**
Sedative use (yes)	**974 (52.25%)**	**351 (61.47%)**	**0.187**	243 (63.12%)	218 (56.62%)	0.133
Albumin use (yes)	205 (11.00%)	70 (12.26%)	0.039	66 (17.14%)	47 (12.21%)	0.14
Vasopressor (yes)	**760 (40.77%)**	**345 (60.42%)**	**0.401**	**231 (60.00%)**	**196 (50.91%)**	**0.184**
Hypertension (yes)	980 (52.58%)	293 (51.31%)	0.025	220 (57.14%)	205 (53.25%)	0.078
Diabetes (yes)	430 (23.07%)	129 (22.59%)	0.011	112 (29.09%)	90 (23.38%)	0.13
Renal disease (yes)	215 (11.53%)	77 (13.49%)	0.059	65 (16.88%)	54 (14.03%)	0.079
Liver disease (yes)	260 (13.95%)	64 (11.21%)	0.083	66 (17.14%)	53 (13.77%)	0.094
COPD (yes)	221 (11.86%)	64 (11.21%)	0.02	35 (9.09%)	47 (12.21%)	0.101
Septic shock (yes)	**487 (26.13%)**	**193 (33.80%)**	**0.168**	**169 (43.90%)**	**124 (32.21%)**	**0.243**
MAP first	82.00 [70.00, 95.00]	83.00 [71.00, 94.00]	0.026			
MAP min	**58.00 [50.00, 65.00]**	**55.00 [47.00, 63.00]**	**0.214**	55.00 [47.00, 62.00]	56.00 [49.00, 64.00]	0.162
MAP max	103.00 [92.00, 118.00]	105.00 [93.00, 120.00]	0.124	101.00 [92.00, 116.00]	104.00 [92.00, 119.00]	0.017
Heart rate first	**93.00 [78.00, 107.00]**	**96.00 [81.00, 111.50]**	**0.103**	—	—	—
Heart rate min	72.00 [61.00, 84.00]	75.00 [61.00, 87.00]	0.068	73.68 (18.71)	73.78 (17.46)	0.006
Heart rate max	108.00 [95.00, 124.00]	110.00 [96.00, 124.00]	0.031	109.00 [95.00, 130.00]	108.00 [96.00, 123.00]	0.135
Temperature first	**36.83 [36.44, 37.28]**	**36.67 [36.06, 37.17]**	**0.314**	—	—	—
Temperature min	**36.50 [36.11, 36.78]**	**36.33 [35.44, 36.67]**	**0.368**	**36.33 [35.56, 36.61]**	**36.44 [35.94, 36.72]**	**0.279**
Temperature max	**37.44 [37.06, 38.11]**	**37.33 [36.89, 38.00]**	**0.248**	37.33 [37.00, 37.89]	37.44 [37.00, 38.06]	0.121
PH first	**7.35 [7.28, 7.41]**	**7.29 [7.19, 7.37]**	**0.458**	—	—	—
PH min	**7.30 [7.22, 7.37]**	**7.22 [7.13, 7.31]**	**0.566**	**7.22 [7.12, 7.32]**	**7.27 [7.17, 7.35]**	**0.325**
PH max	7.44 [7.39, 7.49]	7.44 [7.38, 7.50]	0.098	7.44 [7.38, 7.49]	7.45 [7.40, 7.50]	0.191
PO_2_ first	101.00 [73.00, 161.75]	102.00 [67.75, 174.25]	0.065	—	—	—
PO_2_ min	**70.00 [54.00, 92.00]**	**62.00 [47.00, 80.50]**	**0.257**	**62.00 [46.00, 76.00]**	**65.00 [49.00, 86.00]**	**0.118**
PO_2_ max	**153.00 [102.00, 232.00]**	**181.00 [119.50, 266.00]**	**0.229**	180.00 [115.00, 274.00]	171.00 [111.00, 248.00]	0.07
PCO_2_ first	**41.00 [35.00, 49.00]**	**39.00 [33.00, 50.00]**	**0.095**	—	—	—
PCO_2_ min	**35.00 [30.00, 40.00]**	**32.00 [27.00, 38.00]**	**0.305**	32.00 [28.00, 37.00]	33.00 [28.00, 38.00]	0.092
PCO_2_ max	**47.00 [39.00, 59.00]**	**49.00 [40.00, 62.00]**	**0.099**	49.00 [41.00, 64.00]	48.00 [40.00, 59.00]	0.083
HCO_3_ first	**22.00 [19.00, 25.00]**	**19.00 [16.00, 23.00]**	**0.442**	—	—	—
HCO_3_ min	**20.00 [17.00, 23.00]**	**17.00 [13.00, 20.00]**	**0.567**	**17.00 [13.00, 20.00]**	**19.00 [15.00, 21.00]**	**0.33**
HCO_3_ max	**27.00 [24.00, 31.00]**	**26.00 [22.00, 29.00]**	**0.236**	26.00 [22.00, 30.00]	26.00 [23.00, 30.00]	0.153
Lactate first	**1.90 [1.20, 3.00]**	**3.00 [1.60, 5.53]**	**0.567**	—	—	—
Lactate min	**1.10 [0.80, 1.50]**	**1.20 [0.90, 1.90]**	**0.336**	1.20 [0.90, 1.80]	1.10 [0.90, 1.60]	0.196
Lactate max	**2.20 [1.40, 3.60]**	**3.80 [2.00, 7.70]**	**0.589**	**4.00 [2.20, 7.70]**	**2.70 [1.80, 4.60]**	**0.392**
TNT first	**0.01 [0.01, 0.03]**	**0.19 [0.11, 0.44]**	**0.502**	—	—	—
TNT max	**0.01 [0.01, 0.03]**	**0.25 [0.15, 0.62]**	**0.532**	**0.02 [0.01, 0.05]**	**0.20 [0.13, 0.44]**	**0.521**
BNP first	**996.50 [237.50, 3068.25]**	**1692.00 [758.50, 5805.50]**	**0.288**	—	—	—
BNP max	**1174.00 [240.00, 3288.00]**	**1658.00 [438.00, 7052.00]**	**0.319**	**1748.00 [378.00, 6002.00]**	**1265.00 [257.00, 3966.00]**	**0.15**
Creatinine first	**1.00 [0.70, 1.50]**	**1.40 [1.00, 2.20]**	**0.361**	—	—	—
Creatinine max	**1.20 [0.80, 1.90]**	**2.00 [1.20, 3.60]**	**0.501**	**2.00 [1.20, 3.70]**	**1.50 [1.00, 2.90]**	**0.18**
BUN first	**19.00 [13.00, 32.00]**	**26.00 [17.00, 44.00]**	**0.344**	—	—	—
BUN min	**13.00 [9.00, 22.00]**	**19.00 [12.00, 30.00]**	**0.352**	**19.00 [11.00, 31.00]**	**17.00 [11.00, 28.00]**	**0.118**
BUN max	**27.00 [17.00, 46.00]**	**39.00 [25.00, 68.00]**	**0.435**	**42.00 [26.00, 69.00]**	**36.00 [23.00, 63.00]**	**0.171**
WBC first	**11.60 [7.80, 16.60]**	**13.50 [9.20, 19.50]**	**0.239**	—	—	—
WBC min	7.80 [5.50, 10.80]	8.20 [5.45, 11.80]	0.134	8.10 [4.90, 11.60]	8.00 [5.70, 10.80]	0.068
WBC max	**15.30 [10.80, 21.30]**	**18.80 [12.90, 26.20]**	**0.294**	18.00 [12.60, 25.90]	17.10 [12.40, 24.50]	0.129
HB first	11.00 [9.40, 12.50]	11.10 [9.20, 13.00]	0.079	—	—	—
HB min	**8.90 [7.40, 10.50]**	**8.30 [6.90, 10.20]**	**0.133**	8.00 [6.80, 10.20]	8.30 [6.90, 10.10]	0.071
HB max	**11.40 [10.10, 13.00]**	**11.70 [10.20, 13.40]**	**0.144**	11.70 [10.20, 13.30]	11.40 [9.90, 13.00]	0.109
PLT first	**193.00 [132.00, 262.00]**	**185.00 [125.00, 254.00]**	**0.094**	—	—	—
PLT min	**145.50 [91.00, 206.00]**	**116.00 [69.50, 169.50]**	**0.346**	**104.00 [59.00, 166.00]**	**121.00 [73.00, 181.00]**	**0.17**
PLT max	**243.00 [163.00, 356.00]**	**221.00 [148.50, 318.50]**	**0.153**	218.00 [144.00, 338.00]	226.00 [149.00, 341.00]	0.017
INR first	**1.20 [1.10, 1.50]**	**1.30 [1.10, 1.80]**	**0.179**	—	—	
INR min	**1.20 [1.10, 1.30]**	**1.20 [1.10, 1.40]**	**0.13**	**1.20 [1.10, 1.40]**	**1.10 [1.10, 1.30]**	**0.168**
INR max	**1.40 [1.20, 1.70]**	**1.50 [1.30, 2.30]**	**0.275**	**1.50 [1.30, 2.20]**	**1.40 [1.30, 1.90]**	**0.157**
D-dimer first	**2280.00 [1152.50, 5691.00]**	**4951.00 [2450.00, 8702.00]**	**0.571**	**—**	**—**	**—**
D-dimer min	**1680.00 [1042.50, 4621.00]**	**3169.00 [1286.00, 7012.00]**	**0.357**	**3562.00 [1381.00, 6577.00]**	**2421.00 [1251.00, 6389.00]**	**0.164**
D-dimer max	**4273.00 [1769.00, 8389.00]**	**6765.00 [2321.00, 13978.50]**	**0.269**	6106.00 [2100.00, 14017.00]	5545.00 [2079.00, 9954.00]	0.17
FIB first	**341.00 [187.25, 565.75]**	**266.00 [138.50, 446.50]**	**0.323**	**—**	**—**	**—**
FIB min	**357.00 [203.00, 547.75]**	**284.00 [150.50, 474.00]**	**0.26**	306.00 [159.00, 489.00]	320.00 [185.00, 513.00]	0.068
FIB max	**434.00 [252.00, 674.25]**	**392.00 [226.00, 630.50]**	**0.153**	422.00 [243.00, 649.00]	425.00 [243.00, 649.00]	0.018
PT first	**13.80 [12.40, 16.30]**	**14.50 [12.70, 19.00]**	**0.174**	**—**	**—**	**—**
PT min	12.80 [11.60, 14.40]	12.90 [11.60, 15.35]	0.119	13.00 [11.70, 15.60]	12.80 [11.60, 14.60]	0.159
PT max	**14.80 [13.10, 18.10]**	**16.50 [14.00, 23.90]**	**0.288**	**16.50 [14.50, 23.70]**	**15.70 [13.70, 20.50]**	**0.173**
ALT first	**33.00 [18.00, 69.00]**	**63.00 [28.00, 215.25]**	**0.333**	**—**	**—**	**—**
ALT max	**44.00 [22.00, 104.00]**	**85.00 [34.50, 368.00]**	**0.384**	**90.00 [39.00, 260.00]**	**54.00 [27.00, 134.00]**	**0.254**
AST first	**47.00 [26.00, 97.00]**	**104.50 [49.00, 362.00]**	**0.326**	**—**	**—**	**—**
AST max	**65.00 [32.00, 150.00]**	**134.00 [60.50, 603.50]**	**0.363**	**140.00 [68.00, 471.00]**	**85.00 [47.00, 207.00]**	**0.221**
ABL first	2.90 [2.50, 3.40]	2.90 [2.40, 3.30]	0.116	**—**	**—**	**—**
ABL min	**2.80 [2.30, 3.30]**	**2.70 [2.25, 3.10]**	**0.201**	**2.60 [2.10, 3.00]**	**2.70 [2.30, 3.10]**	**0.248**
ABL max	3.10 [2.70, 3.50]	3.00 [2.70, 3.50]	0.076	3.00 [2.60, 3.50]	3.10 [2.70, 3.50]	0.131
Ca first	**8.10 [7.50, 8.70]**	**8.00 [7.40, 8.60]**	**0.056**	**—**	**—**	**—**
Ca min	**7.60 [7.10, 8.10]**	**7.40 [6.90, 7.90]**	**0.19**	**7.40 [7.00, 7.80]**	**7.50 [7.00, 8.00]**	**0.132**
Ca max	8.80 [8.30, 9.30]	8.80 [8.30, 9.50]	0.116	8.90 [8.30, 9.60]	8.80 [8.30, 9.40]	0.065
Na first	**139.00 [135.00, 142.00]**	**139.00 [136.00, 142.00]**	**0.105**	**—**	**—**	**—**
Na min	136.00 [132.00, 138.00]	135.00 [132.00, 138.00]	0.019	134.00 [131.00, 138.00]	135.00 [132.00, 138.00]	0.037
Na max	**143.00 [140.00, 147.00]**	**144.00 [141.00, 149.00]**	**0.17**	144.00 [140.00, 149.00]	144.00 [140.00, 149.00]	0.033
K first	**4.10 [3.70, 4.60]**	**4.20 [3.70, 4.90]**	**0.19**	**—**	**—**	**—**
K min	3.40 [3.10, 3.70]	3.40 [3.10, 3.70]	0.015	3.30 [3.00, 3.70]	3.40 [3.10, 3.70]	0.033
K max	**4.60 [4.20, 5.20]**	**5.00 [4.50, 5.80]**	**0.316**	**5.00 [4.50, 5.80]**	**4.80 [4.40, 5.50]**	**0.202**
CRP first	99.70 [38.60, 204.40]	99.30 [38.60, 217.50]	0.023	**—**	**—**	**—**
CRP max	129.80 [58.95, 218.15]	120.10 [66.00, 230.35]	0.026	103.20 [45.80, 210.80]	99.70 [47.40, 220.40]	0.001
CKMB first	**4.00 [2.00, 7.25]**	**13.50 [6.00, 31.00]**	**0.545**	**—**	**—**	**—**

COPD, chronic obstructive pulmonary disease; MAP, mean arterial pressure; PH, power of hydrogen; PO_2_, partial pressure of oxygen; PCO_2_, partial pressure of carbon dioxide; TNT, troponin T; BNP, brain natriuretic peptide; BUN, blood urea nitrogen; WBC, white blood cell; HB, hemoglobin; PLT, platelet; INR, international normalized ratio; FIB, fibrinogen; PT, prothrombin time; ALT, alanine aminotransferase; AST, aspartate aminotransferase; ABL, albumin; Ca, calcium ion; Na, sodium ion; K, potassium ion; CRP, c-reactive protein; CKMB, creatine kinase MB; SIMI, sepsis-induced myocardial injury. Values are presented as mean (standard deviation) or median [Q1, Q3] for continuous variables and number (percentage) for categorical variables. Variables in bold have *p*-value <0.05.

### Feature selection

**Figure 2:**
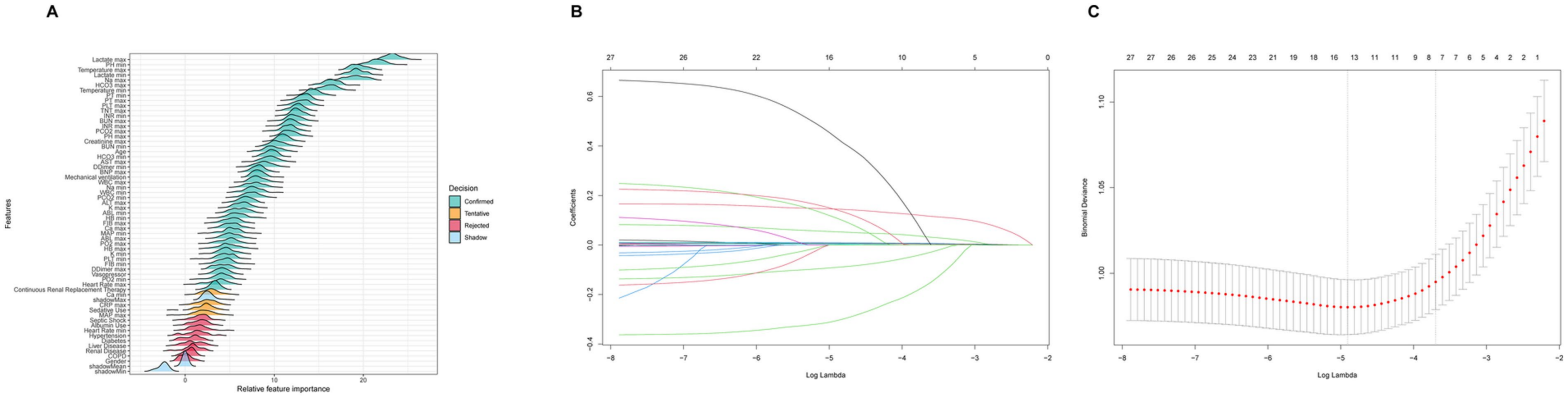
**(A)** Boruta variable selection for prognostic analysis section. **(B)** The optimal parameter (lambda) for the least absolute shrinkage and selection operator (LASSO) model was determined through 10-fold cross-validation employing the minimum criteria. The partial likelihood deviance (binomial deviance) curve was depicted against the log(lambda). **(C)** The coefficient profiles from the LASSO model. A plot of the coefficient profiles was created against the log(*λ*) sequence.

In the prognostic analysis segment, we implemented Boruta variable selection and ultimately incorporated 46 variables into the prognostic analysis, as illustrated in Figure 2A. Supplementary Figure S1 highlights that the most significant covariates distinguishing the non-SIMI and SIMI groups. In the ML section, we utilized LASSO variable selection to construct a more parsimonious model. We selected a lambda of 0.057300 at one standard error, which resulted in eight variables [creatine kinase-myocardial band (CKMB) first, creatinine first, alanine aminotransferase (ALT) first, lactate first, blood urea nitrogen (BUN) first, temperature first, vasopressor use, and liver disease] for building the ML model, as illustrated in Figures 2B,C.

### Outcomes and sensitivity studies

IPTW, based on the estimated propensity scores, was employed to standardize differences between the non-SIMI and SIMI groups. Detailed results are provided in Table 1 and Supplementary Figure S2. Leveraging a doubly robust analysis employing a survey-weighted GLM/Cox model adjusted with Boruta-selected covariates and using IPTW as the primary focus, we found that patients with SIMI exhibited significantly worse short- and intermediate-term prognosis compared to those without SIMI. However, the one-year all-cause mortality risk showed no significant difference [in-ICU mortality: odds ratio (OR) 1.38, 95% CI 1.02–1.86, *p* < 0.05; 28-day mortality: OR 1.35, 95% CI 1.02–1.79, *p* < 0.05; 180-day mortality: HR 1.21, 95% CI 1.01–1.44, *p* < 0.05; 1-year mortality: HR 1.03, 95% CI 0.99–1.08, *p* = 0.169]. These findings were robustly validated through comprehensive sensitivity analyses across diverse estimation models, as detailed in Table 2; Supplementary Tables S4–S24 and Supplementary Figures S3–S5. The covariates adjusted in different models are detailed in the Supplementary material.

**Table 2:** Primary outcome with different models for cohort.

	Result	*p*-value
In-ICU mortality		
Log-rank test [HR (95% CI)]	**2.51 (2.04, 3.09)**	**<0.001**
Multivariate logistic model adjusted with Boruta selected covariates [OR (95% CI)]	**1.55 (1.15, 2.09)**	**<0.01**
Multivariate logistic model adjusted with unbalanced covariates [OR (95% CI)]	**1.52 (1.13, 2.03)**	**<0.01**
Logistic model adjusted with Boruta selected covariates using IPTW [HR (95% CI)]	**1.38 (1.12, 1.69)**	**<0.01**
Logistic model adjusted with unbalanced covariates using IPTW [HR (95% CI)]	**1.32 (1.08, 1.61)**	**<0.01**
Survey-weighted GLM model adjusted with Boruta selected covariates using IPTW [OR (95% CI)]	**1.38 (1.02, 1.86)**	**<0.05**
28-day mortality		
Log-rank test [HR (95% CI)]	**2.60 (2.15, 3.16)**	**<0.001**
Multivariate logistic model adjusted with Boruta selected covariates [OR (95% CI)]	**1.48 (1.14, 1.93)**	**<0.01**
Multivariate logistic model adjusted with unbalanced covariates [OR (95% CI)]	**1.45 (1.11, 1.88)**	**<0.01**
Logistic model adjusted with Boruta selected covariates using IPTW [HR (95% CI)]	**1.35 (1.14, 1.61)**	**<0.001**
Logistic model adjusted with unbalanced covariates using IPTW [HR (95% CI)]	**1.3 (1.10, 1.53)**	**<0.01**
Survey-weighted GLM model adjusted with Boruta selected covariates using IPTW [OR (95% CI)]	**1.35 (1.02, 1.79)**	**<0.05**
180-day mortality		
Log-rank test [HR (95% CI)]	**2.39 (2.01, 2.84)**	**<0.001**
Multivariate Cox model adjusted with Boruta selected covariates [HR (95% CI)]	**1.29 (1.10, 1.52)**	**<0.01**
Multivariate Cox model adjusted with unbalanced covariates [HR (95% CI)]	**1.28 (1.09, 1.50)**	**<0.01**
Cox model adjusted with Boruta selected covariates using IPTW [HR (95% CI)]	**1.2 (1.01, 1.44)**	**<0.05**
Cox model adjusted with unbalanced covariates using IPTW [HR (95% CI)]	**1.17 (1.00, 1.35)**	**<0.05**
Survey-weighted Cox model adjusted with Boruta selected covariates using IPTW [HR (95% CI)]	**1.21 (1.01, 1.44)**	**<0.05**
1-year mortality		
Log-rank test [HR (95% CI)]	**2.24 (1.89, 2.64)**	**<0.001**
Multivariate Cox model adjusted with Boruta selected covariates [HR (95% CI)]	**1.04 (1.00, 1.09)**	**<0.05**
Multivariate Cox model adjusted with unbalanced covariates [HR (95% CI)]	1.04 (1.00, 1.08)	0.079
Cox model adjusted with Boruta selected covariates using IPTW [HR (95% CI)]	1.15 (0.97, 1.37)	0.107
Cox model adjusted with unbalanced covariates using IPTW [HR (95% CI)]	1.12 (0.95, 1.33)	0.187
Survey-weighted Cox model adjusted with Boruta selected covariates using IPTW [HR (95% CI)]	1.03 (0.99, 1.08)	0.169

HR, Hazard ratio; OR, odds ratio; CI, confidence interval; IPTW, inverse probability of treatment weighting; GLM, generalized linear model. Statistical analyses of different models with *p*-value <0.05 were displayed in bold.

### Model evaluation and comparison

**Figure 3:**
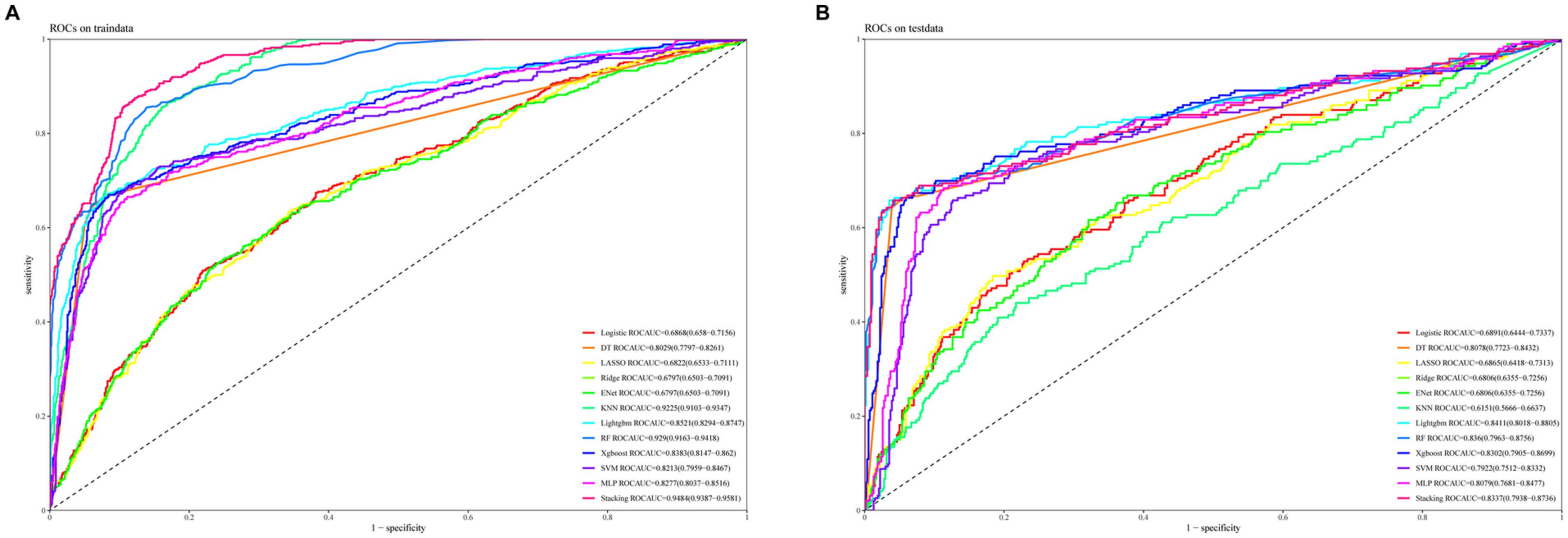
ROC curves for the test and validation sets of 12 machine learning models are shown. **(A)** ROC curves for the training set. **(B)** ROC curves for the test set.

**Figure 4:**
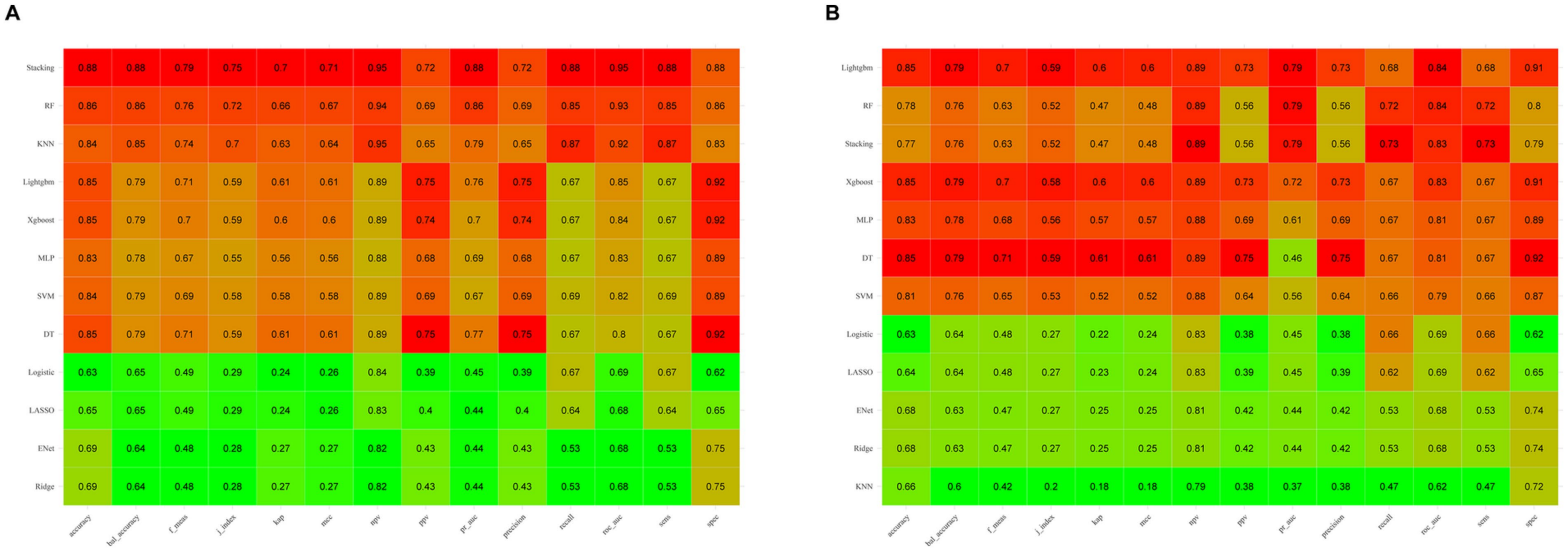
Comparison of machine learning model performance on the training **(A)** and test **(B)** sets across various evaluation metrics. Higher values are highlighted in red, whereas lower values are shown in green, illustrating the models’ effectiveness in both sets.

**Figure 5:**
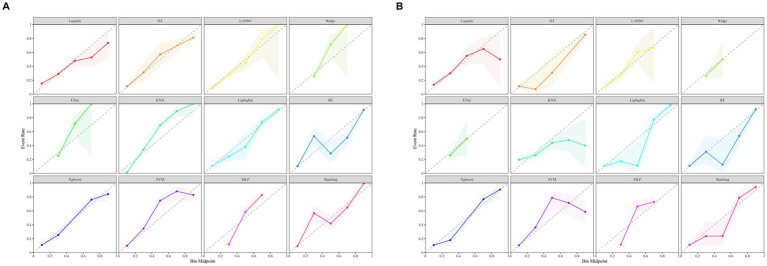
Calibration curves of 12 machine learning models. **(A)** Calibration curves for the training set. **(B)** Calibration curves for the test set.

Figure 3A illustrates the AUROC curves for the training set of 12 ML models, while Figure 3B displays the AUROC curves for the test set. In the test set, the XGBoost model emerged as the optimal predictor, demonstrating superior performance with an AUROC of 0.83 (95% CI: 0.79–0.87), indicating exceptional predictive capabilities. Consequently, the interpretability analysis of the XGBoost model became the primary focus of subsequent investigations. Figure 4 comprehensively depicts the performance of various ML models across both training (A) and test (B) datasets. The calibration curves for the training and test sets of each model are shown in Figure 5, demonstrating that although the AUROC of the XGBoost model is not the highest, it is only 0.01 lower than the highest value. Moreover, the calibration curve of the XGBoost model is closer to the ideal diagonal line, indicating that it has greater accuracy and reliability compared with other models.

### Feature importance in XGBoost models

**Figure 6:**
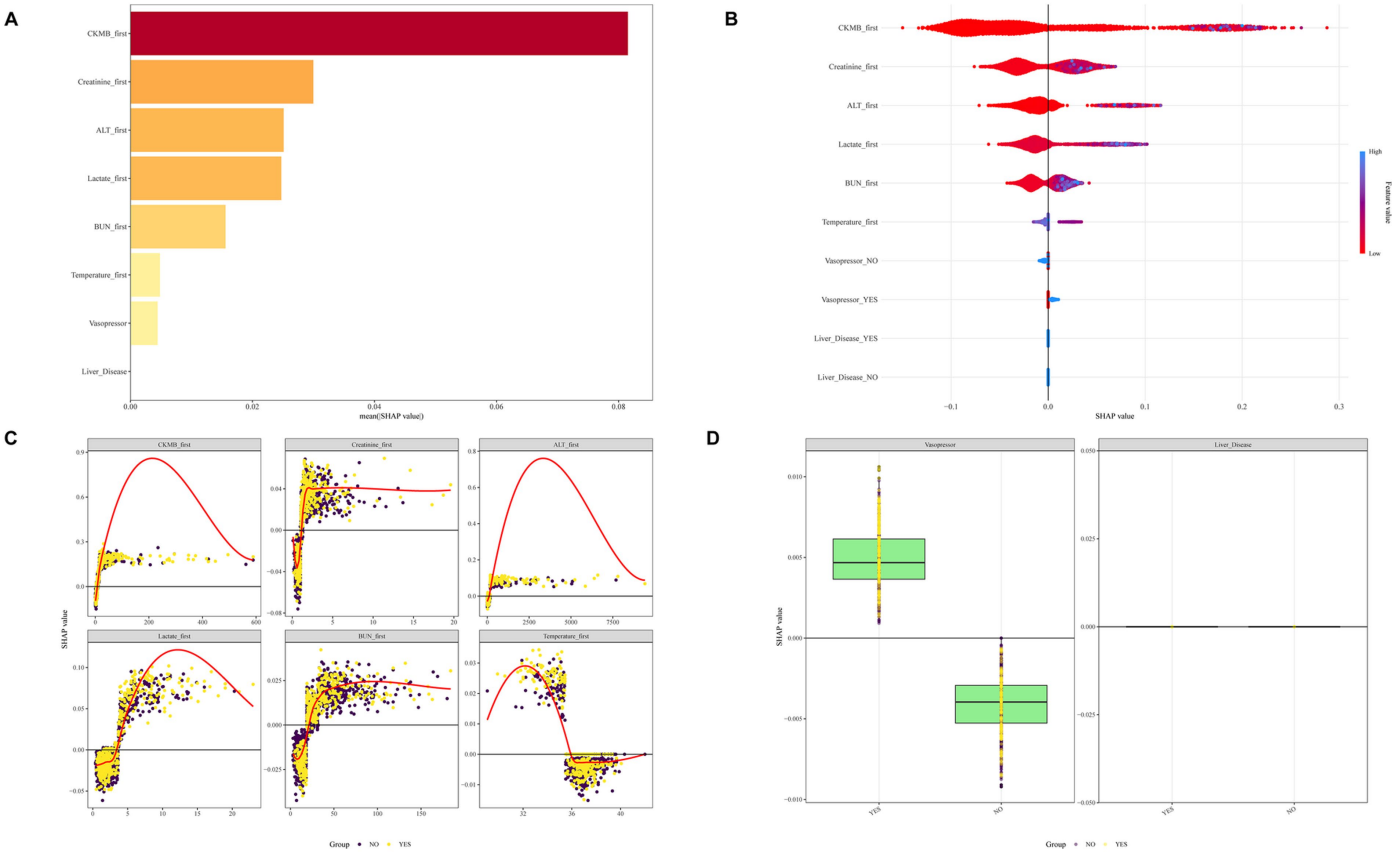
SHAP diagrams of the XGBoost model. **(A)** Ranking of variables by SHAP values. **(B)** SHAP honeycomb diagram. **(C)** Trend diagram of SHAP value changes for continuous variables. **(D)** Box plot of SHAP values for categorical variables.

We conducted SHAP analysis to evaluate the significance of individual characteristic variables within the XGBoost model and their predictive contributions, with results presented in Figures 6A,B. The analysis consistently highlighted CKMB as the most critical variable, exhibiting the largest SHAP value and emerging as a pivotal risk factor for SIMI. Subsequent influential variables in descending order included creatinine, ALT, lactate, BUN, temperature, vasopressor use, and liver disease. The study further explored subgroup-specific outcomes for continuous and categorical variables in populations with and without SIMI (Figures 6C,D), providing comprehensive insights into variable interactions and predictive mechanisms.

### Model application development

**Figure 7:**
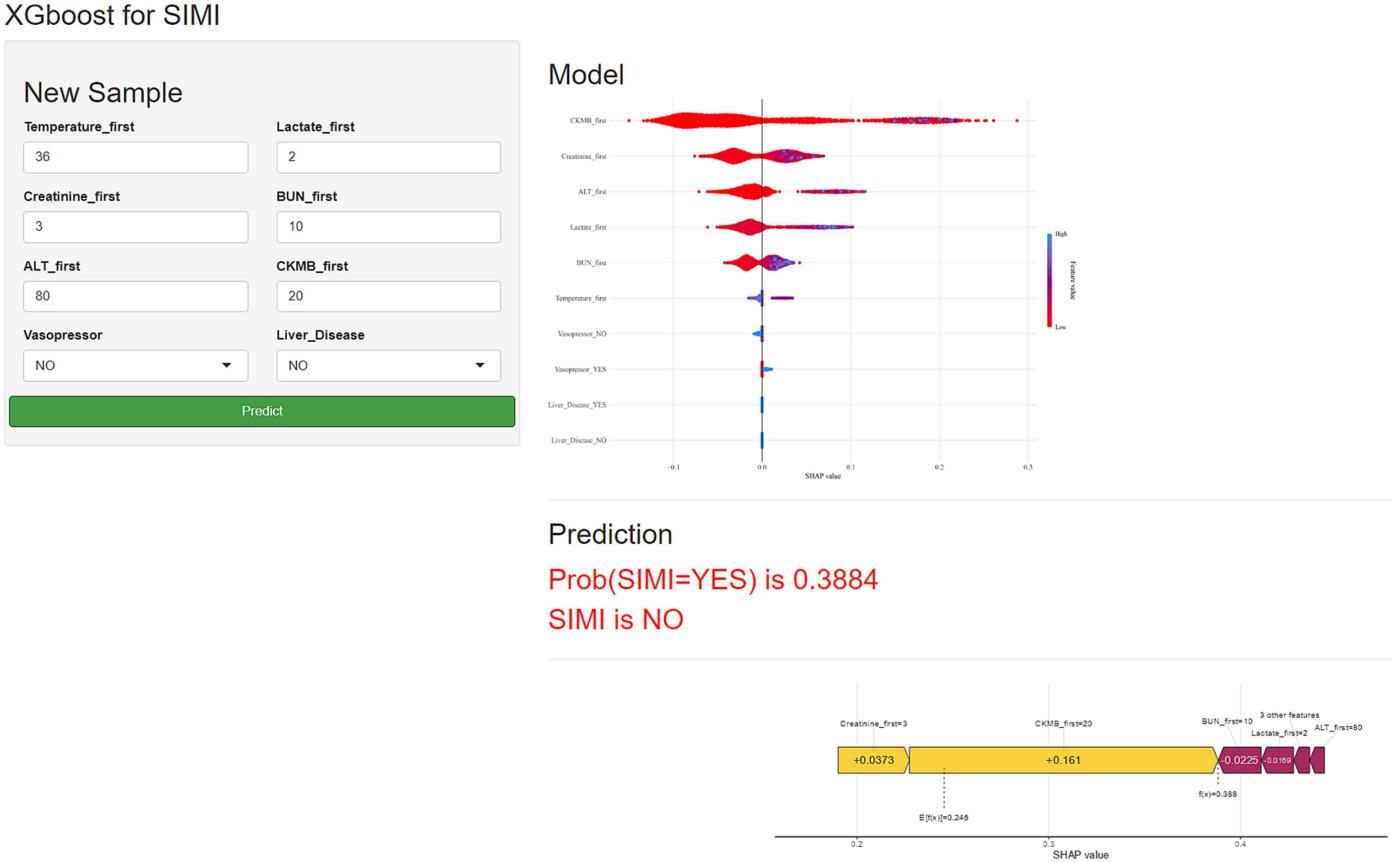
Schematic diagram of the web-based application for predicting sepsis-induced myocardial injury.

To enhance the generalizability and clinical utility of our predictive model for SIMI, we developed a comprehensive web-based application designed for external validation and real-world implementation. The platform, accessible at https://qhdpanguo.shinyapps.io/SIMI/, represents a critical translation of our ML algorithm into a user-friendly, interactive clinical tool (Figure 7).

## Discussion

In this study, we conducted a comprehensive assessment of patients with SIMI. We demonstrated that SIMI diagnosis can be simplified and made more clinically applicable, achievable through TNT results alone. Prognostic analysis revealed that SIMI patients exhibit significantly worse short- and medium-term outcomes compared to non-SIMI patients. Therefore, early identification of high-risk patients for in-hospital SIMI development is of paramount importance, which motivated our construction of a ML model for predicting in-hospital SIMI risk. To the best of our knowledge, this is the first study to develop a ML model for such patients and create a web-based application for external validation and clinical application. This approach can assist clinicians in the early identification of high-risk SIMI patients and enable timely interventions.

Currently, there is no clear consensus on the definition of SIMI. However, most studies apply the Sepsis-3.0 criteria to determine sepsis occurrence and then exclude patients with other conditions that could elevate troponin levels. These patients are then diagnosed with SIMI based on their troponin values (13, 14). The threshold for troponin levels in SIMI diagnosis remains controversial. Some studies utilizing the MIMIC database emphasize that TNT ≥0.01 ng/mL should define SIMI (14, 15). However, our attempt to extract records with TNT <0.01 ng/mL from the MIMIC database yielded only 24 records, making it implausible that the vast majority of hundreds of thousands of hospitalized patients would exhibit myocardial injury. Authoritative research supports TNT ≥0.1 ng/mL as positive (16), and the MIMIC database explicitly states that TNT ≥0.1 ng/mL is required to determine myocardial injury. Therefore, we adhere to the original laboratory indicator in the MIMIC database, using TNT ≥0.1 ng/mL as the criterion for SIMI diagnosis. We chose the maximum TNT value after ICU admission as the diagnostic standard, consistent with previous studies (17), to facilitate the identification of more SIMI patients. Subsequently, we explored the impact of our diagnostic criteria on patient outcomes. Results demonstrated that patients diagnosed with SIMI had significantly worse short- and medium-term prognoses compared to non-SIMI patients, thus confirming the clinical significance of this diagnosis.

Numerous studies have consistently demonstrated the poor prognosis of SIMI patients. Garcia et al. (17) conducted a large-scale, multicenter retrospective study revealing a significant correlation between troponin elevation during acute infection and substantially increased risks of major adverse cardiovascular events (MACE) within the subsequent 12-month period. These events encompassed acute myocardial infarction, cerebrovascular accidents, *de novo* atrial fibrillation, and decompensated heart failure, highlighting the critical prognostic value of troponin as a long-term cardiovascular risk stratification biomarker. Angriman et al. (18) investigated 268,259 adult sepsis patients without pre-existing cardiovascular disease, demonstrating a significantly elevated risk of major cardiovascular events during a median follow-up of 3 years. Liang et al. (19) utilized the MIMIC-III database to reveal that sepsis-induced cardiomyopathy (SIC) patients exhibited higher in-hospital mortality compared to non-SIC patients. Consistent with these findings, our research concluded that SIMI patients significantly experienced elevated risks of in-ICU mortality, 28-day mortality, and 180-day mortality compared to non-SIMI patients.

To predict the risk of SIMI earlier, we constructed up to 12 ML models using initial admission assessment data from various indicators and validated them. The validation results suggested that the XGBoost model performed best on the test set, with an ROC AUC of 0.83, accuracy of 0.85, NPV of 0.89, PPV of 0.73, sensitivity of 0.67, and specificity of 0.91. These outstanding metrics led us to select the XGBoost model for further analysis.

Subsequently, we performed SHAP analysis on the model, revealing the top five predictive features: CKMB, creatinine, ALT, lactate and BUN. CKMB, a well-established biomarker of myocardial damage, demonstrates earlier elevation compared to TNT and exhibits significant predictive potential for SIMI. Moreover, CKMB serves as a valuable prognostic indicator for assessing complications and mortality risk (20, 21). Creatinine is an indicator of renal function. As sepsis severity increases, the risk of acute kidney injury (AKI) escalates (22). The underlying pathological mechanisms include direct inflammatory factor-induced tubular cell damage (23), sepsis-associated hypotension and renal hypoperfusion (24), and metabolic reprogramming and adaptive responses of tubular cells leading to AKI (25). ALT is an important indicator of liver function. An acute elevation in ALT represents a rapid deterioration of liver function. Plasma ALT levels can be used for early diagnosis of sepsis-related liver injury (26), and its elevation is significantly associated with the prognosis of patients with sepsis (27, 28). Lactate represents a significant clinical concern, especially in sepsis, serving as a marker of tissue hypoperfusion and metabolic derangement (29). Lactate accumulation indicates a shift towards anaerobic metabolism due to inadequate tissue oxygenation, a characteristic of septic shock (30). In critically ill patients, elevated lactate levels are associated with increased morbidity and mortality, making it a crucial parameter for monitoring and management in intensive care settings (31). BUN, primarily excreted by the kidneys, is the main end product of protein metabolism. BUN levels elevate under two conditions: increased protein catabolism or decreased glomerular filtration rate. In sepsis patients, both conditions frequently occur—there is enhanced protein breakdown and a high risk of acute renal injury, leading to elevated BUN levels (32, 33).

We developed the model into a web-based application and released it, facilitating further validation and dissemination of the model. Additionally, the application allows for dynamic adjustments to the model based on the incorporation of new data.

### Limitations

This study has three limitations. First, it is a single-center retrospective study, which may limit the generalizability of the findings to broader populations. Second, the ML model requires further external validation to confirm its robustness and applicability in diverse clinical settings. Thirdly, our study conducted an analysis on the overall ICU patient population without detailed stratification of different ICU subtypes or primary diagnoses. While our model demonstrates good discriminatory power for SIMI patients, it may not adequately account for the high dimensionality and heterogeneity typical of clinical data (34). Future research could benefit from a more granular analysis, considering the unique characteristics and risk factors associated with different ICU environments and patient populations, thereby enhancing the model’s precision and applicability across diverse clinical settings.

## Conclusion

In conclusion, the development of SIMI significantly worsens patient prognosis, and ML models have proven to be reliable tools for its prediction. Among all the predictive models, the XGBoost model has exhibited the highest effectiveness, resulting in the development of an externally applicable application based on this model. This application may support clinicians in providing precise management and initiating timely interventions for septic patients at risk of SIMI, ultimately helping to reduce mortality.

## Data Availability

The datasets presented in this article are not readily available because access to the MIMIC dataset is restricted and requires principal investigator authorization; therefore, the data cannot be publicly shared. Requests to access the datasets should be directed to https://www.physionet.org/content/mimiciv/3.0/.

## Footnotes

^1^ www.shinyapps.io
