# Supplementary material for: Prognostic analysis of sepsis-induced myocardial injury patients using propensity score matching and doubly robust analysis with machine learning-based risk prediction model development
Source: Front Med (Lausanne). 2025 Feb 19;12:1555103. doi: 10.3389/fmed.2025.1555103 (PMC11880261; doi:10.3389/fmed.2025.1555103)
Supplement: Supplementary file 1 [file Data_Sheet_1.docx]

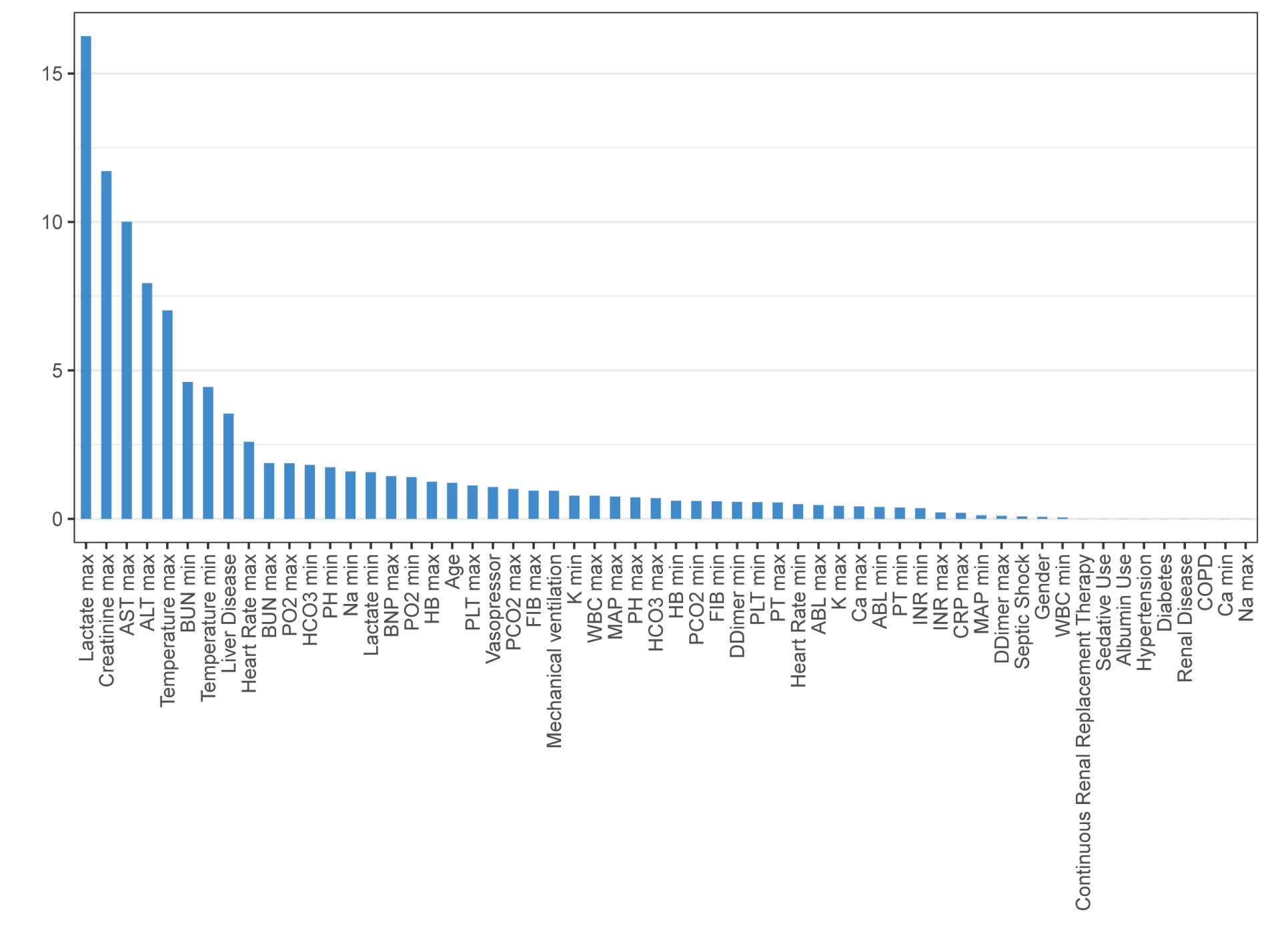


**Figure S1.** The relative influence factor evaluates the discriminative power of 49 covariates within the propensity score model in predicting the probability of sepsis-induced myocardial injury


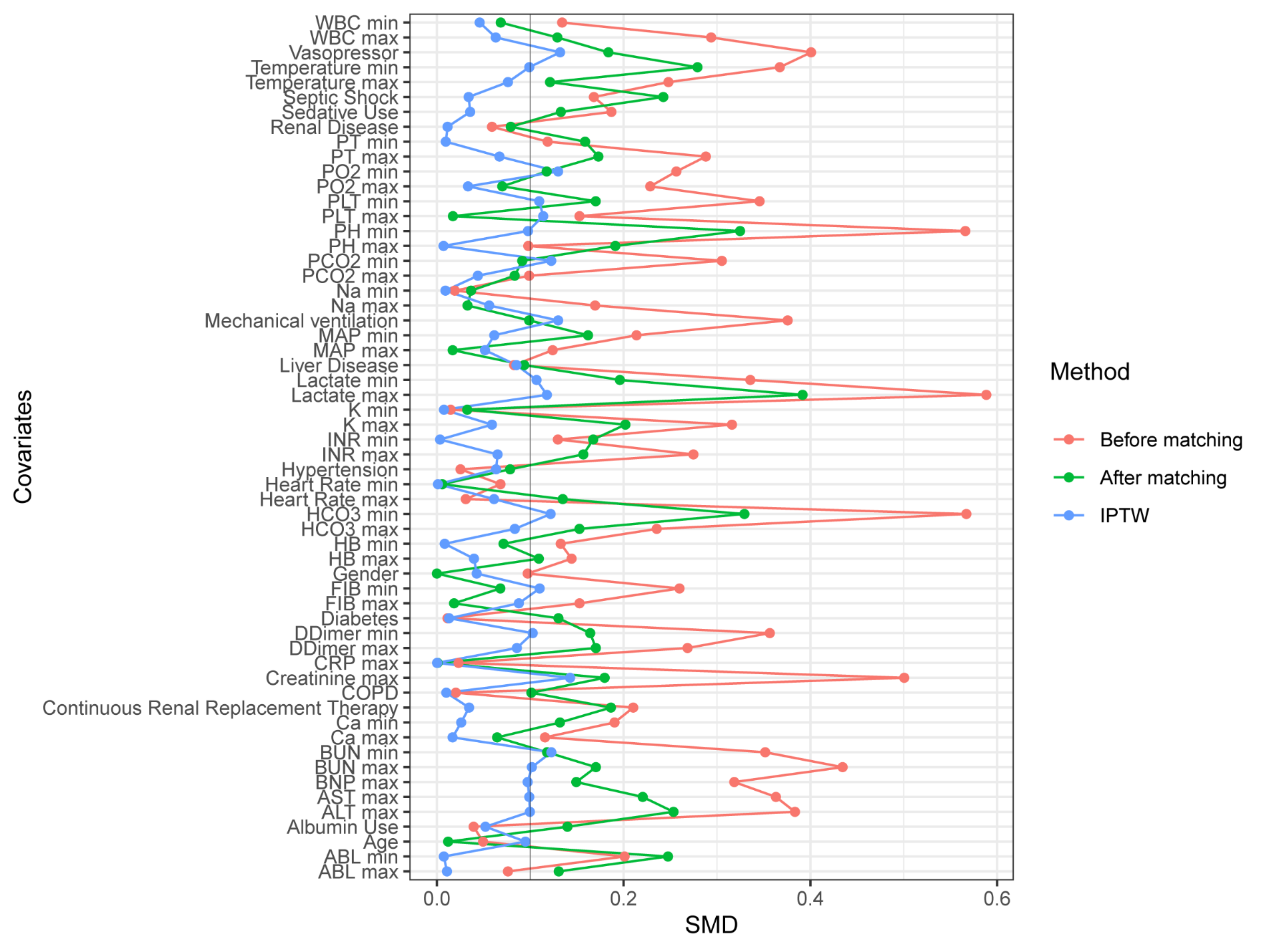


**Figure S2.** Change in standardized mean difference (SMD) of cohort.


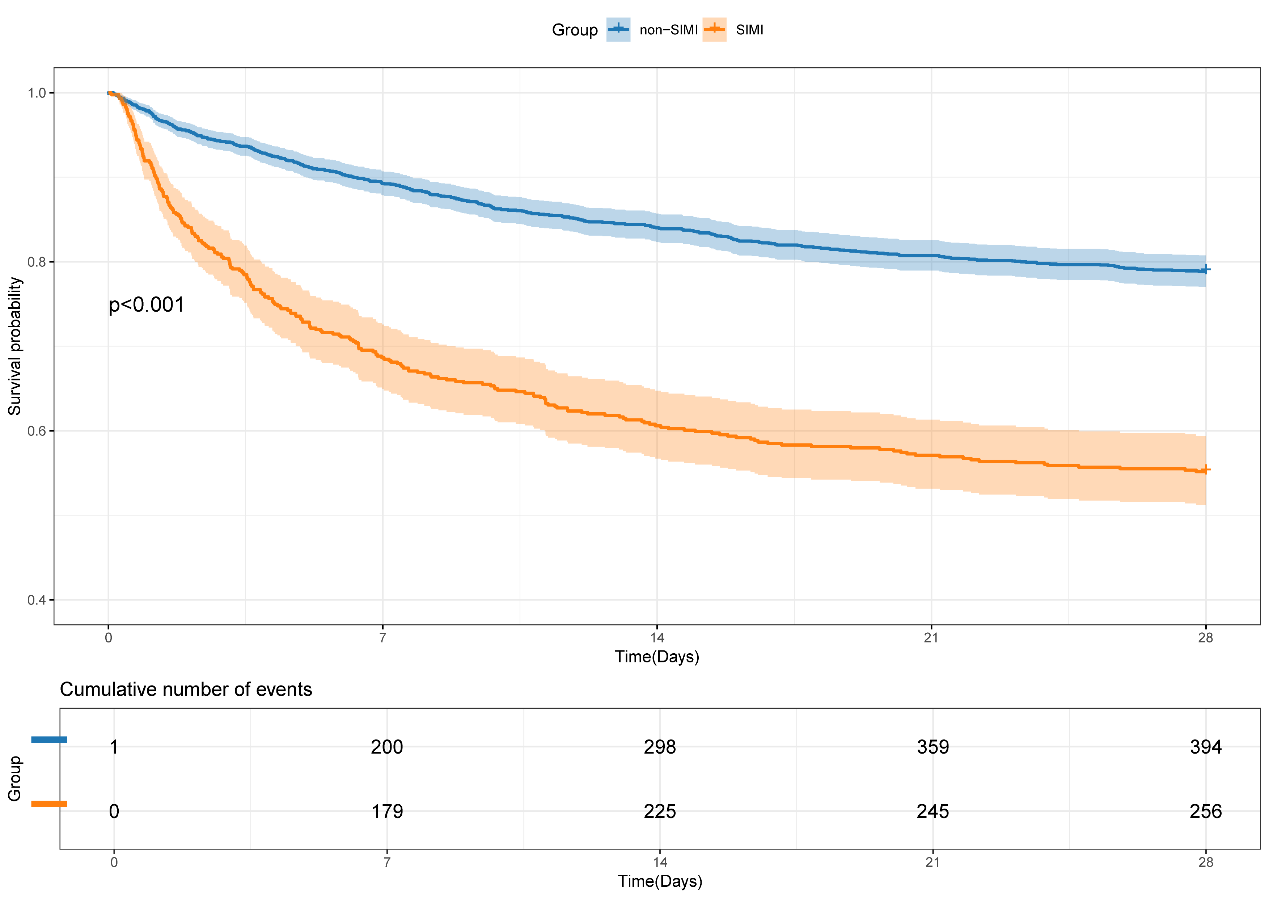


**Figure S3.** Unadjusted Kaplan-Meier survival curve for 28-day mortality


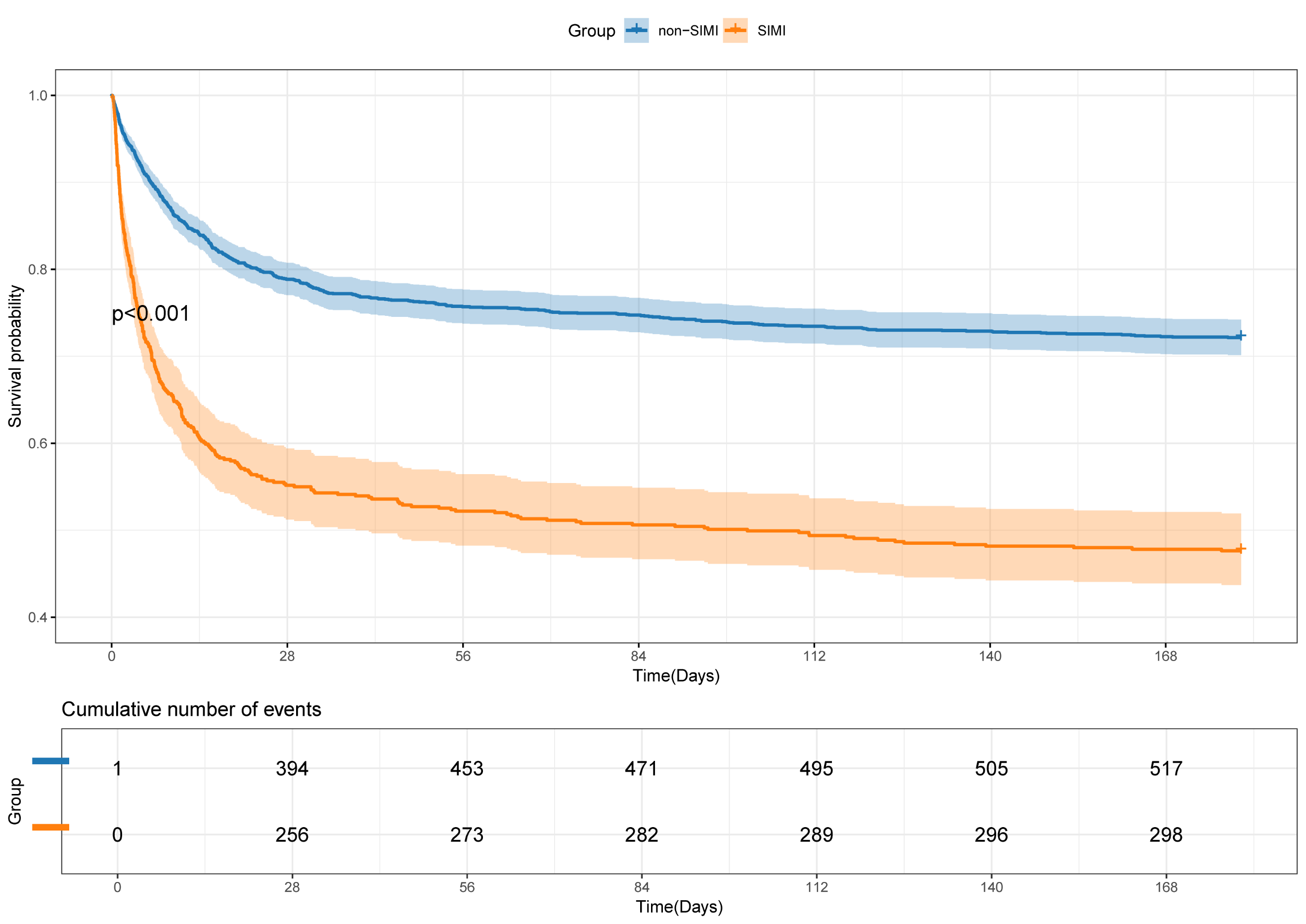


**Figure S4.** Unadjusted Kaplan-Meier survival curve for 180-day mortality


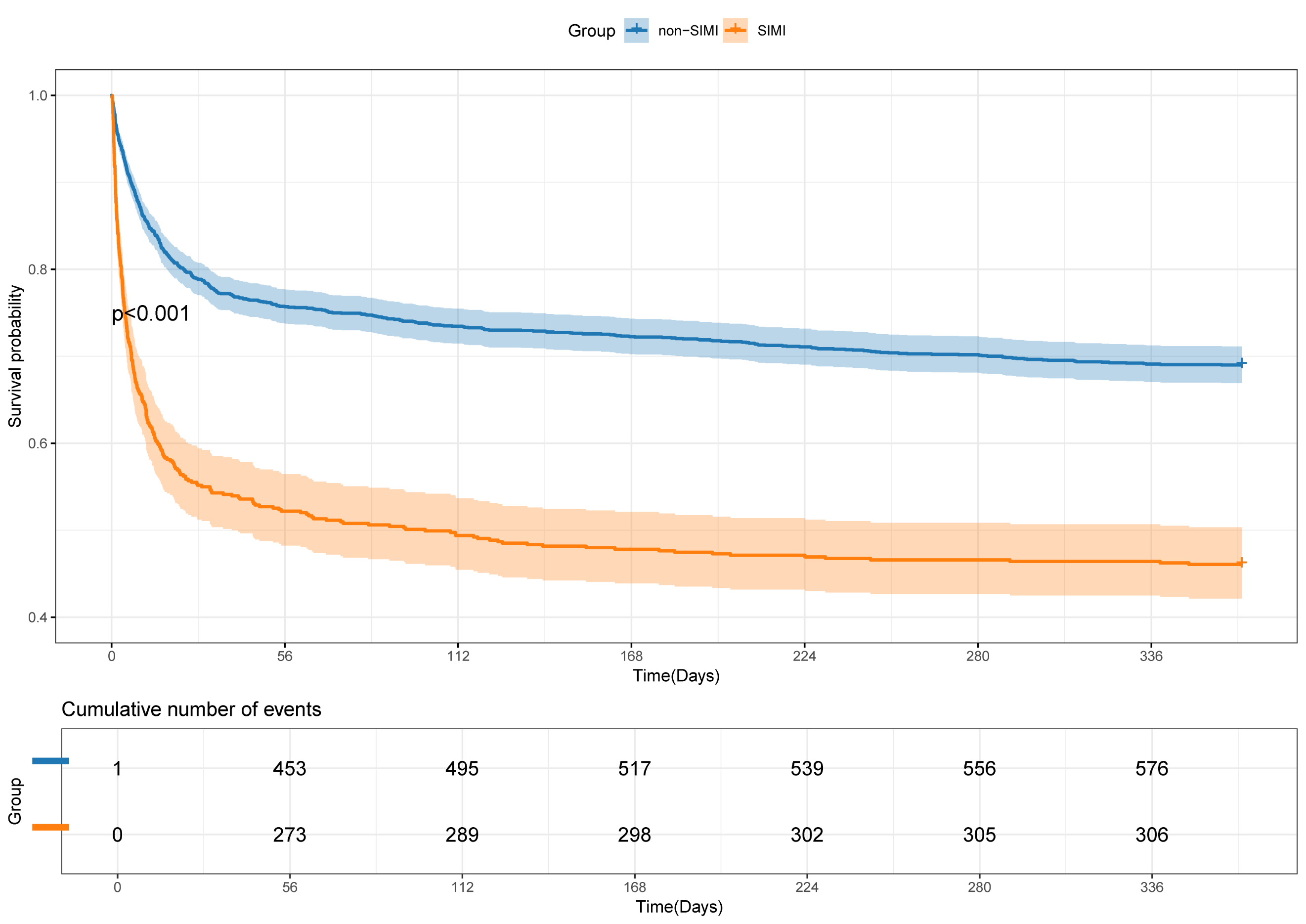


**Figure S5.** Unadjusted Kaplan-Meier survival curve for 1-year mortality
