# Supplementary material for: Prognostic analysis of sepsis-induced myocardial injury patients using propensity score matching and doubly robust analysis with machine learning-based risk prediction model development
Source: Front Med (Lausanne). 2025 Feb 19;12:1555103. doi: 10.3389/fmed.2025.1555103 (PMC11880261; doi:10.3389/fmed.2025.1555103)
Supplement: Supplementary file 2 [file Data_Sheet_2.docx]

Supplementary Table 1. Basic demographic characteristics of the original cohort

|  | **non-SIMI (N=1864)** | **SIMI (N=571)** | **p-value** | **SMD** |
| --- | --- | --- | --- | --- |
| Age | 61.00 [50.00, 74.00] | 63.00 [51.00, 75.00] | 0.294 | 0.049 |
| Gender (Female) | **847 (45.44%)** | **232 (40.63%)** | **<0.05** | **0.097** |
| Mechanical ventilation (YES) | **1024 (54.94%)** | **415 (72.68%)** | **<0.001** | **0.376** |
| Continuous Renal Replacement Therapy (YES) | **69 (3.70%)** | **50 (8.76%)** | **<0.001** | **0.21** |
| Sedative Use (YES) | **974 (52.25%)** | **351 (61.47%)** | **<0.001** | **0.187** |
| Albumin Use (YES) | 205 (11.00%) | 70 (12.26%) | 0.449 | 0.039 |
| Vasopressor (YES) | **760 (40.77%)** | **345 (60.42%)** | **<0.001** | **0.401** |
| Hypertension (YES) | 980 (52.58%) | 293 (51.31%) | 0.631 | 0.025 |
| Diabetes (YES) | 430 (23.07%) | 129 (22.59%) | 0.857 | 0.011 |
| Renal Disease (YES) | 215 (11.53%) | 77 (13.49%) | 0.237 | 0.059 |
| Liver Disease (YES) | 260 (13.95%) | 64 (11.21%) | 0.106 | 0.083 |
| COPD (YES) | 221 (11.86%) | 64 (11.21%) | 0.729 | 0.02 |
| Septic Shock (YES) | **487 (26.13%)** | **193 (33.80%)** | **<0.001** | **0.168** |
| MAP min | **58.00 [50.00, 65.00]** | **55.00 [47.00, 63.00]** | **<0.001** | **0.214** |
| MAP max | 103.00 [92.00, 118.00] | 105.00 [93.00, 120.00] | 0.086 | 0.124 |
| Heart Rate min | 72.00 [61.00, 84.00] | 75.00 [61.00, 87.00] | 0.111 | 0.068 |
| Heart Rate max | 108.00 [95.00, 124.00] | 110.00 [96.00, 124.00] | 0.332 | 0.031 |
| Temperature min | **36.50 [36.11, 36.78]** | **36.33 [35.45, 36.67]** | **<0.001** | **0.365** |
| Temperature max | **37.44 [37.06, 38.11]** | **37.31 [36.89, 38.00]** | **<0.001** | **0.246** |
| PH min | **7.29 [7.21, 7.36]** | **7.22 [7.12, 7.30]** | **<0.001** | **0.552** |
| PH max | 7.45 [7.39, 7.50] | 7.45 [7.38, 7.50] | 0.33 | 0.123 |
| PO2 min | **66.00 [52.00, 86.00]** | **59.00 [45.00, 78.00]** | **<0.001** | **0.193** |
| PO2 max | **168.00 [114.00, 245.00]** | **194.00 [135.25, 298.00]** | **<0.001** | **0.277** |
| PCO2 min | **34.00 [29.00, 39.00]** | **31.00 [26.00, 36.00]** | **<0.001** | **0.366** |
| PCO2 max | 50.00 [41.00, 63.00] | 51.00 [41.00, 64.00] | 0.577 | 0.024 |
| HCO3 min | **20.00 [17.00, 23.00]** | **17.00 [13.00, 20.00]** | **<0.001** | **0.566** |
| HCO3 max | **27.00 [24.00, 31.00]** | **26.00 [22.00, 29.00]** | **<0.001** | **0.231** |
| Lactate min | **1.10 [0.80, 1.50]** | **1.20 [0.90, 1.90]** | **<0.001** | **0.336** |
| Lactate max | **2.35 [1.50, 3.90]** | **4.10 [2.10, 8.20]** | **<0.001** | **0.587** |
| TNT max | **0.01 [0.01, 0.03]** | **0.25 [0.15, 0.62]** | **<0.001** | **0.532** |
| BNP max | **1036.00 [242.50, 3343.00]** | **1741.00 [989.25, 7222.25]** | **<0.01** | **0.312** |
| Creatinine max | **1.20 [0.80, 1.90]** | **2.00 [1.20, 3.60]** | **<0.001** | **0.498** |
| BUN min | **13.00 [9.00, 22.00]** | **19.00 [12.00, 30.00]** | **<0.001** | **0.351** |
| BUN max | **27.00 [17.00, 46.00]** | **39.00 [25.00, 68.00]** | **<0.001** | **0.43** |
| WBC min | 7.70 [5.50, 10.80] | 8.20 [5.40, 11.70] | 0.104 | 0.129 |
| WBC max | **15.30 [10.80, 21.30]** | **18.80 [12.90, 26.22]** | **<0.001** | **0.296** |
| HB min | **8.90 [7.40, 10.50]** | **8.30 [6.90, 10.20]** | **<0.001** | **0.143** |
| HB max | **11.40 [10.10, 13.00]** | **11.70 [10.20, 13.40]** | **<0.05** | **0.143** |
| PLT min | **145.00 [91.00, 206.00]** | **115.00 [69.00, 168.00]** | **<0.001** | **0.348** |
| PLT max | **242.00 [163.00, 354.00]** | **221.00 [149.00, 319.50]** | **<0.01** | **0.145** |
| INR min | **1.15 [1.10, 1.30]** | **1.20 [1.10, 1.40]** | **<0.05** | **0.131** |
| INR max | **1.40 [1.20, 1.70]** | **1.50 [1.30, 2.30]** | **<0.001** | **0.273** |
| DDimer min | **1515.00 [958.50, 3709.00]** | **3692.00 [1381.00, 7262.00]** | **<0.001** | **0.566** |
| DDimer max | **4218.50 [1726.50, 8510.25]** | **7262.00 [3660.00, 15280.00]** | **<0.01** | **0.373** |
| FIB min | **305.00 [157.00, 500.75]** | **219.00 [120.50, 392.00]** | **<0.001** | **0.368** |
| FIB max | **431.00 [243.00, 675.00]** | **372.00 [211.00, 608.00]** | **<0.01** | **0.213** |
| PT min | 12.80 [11.60, 14.40] | 12.90 [11.60, 15.40] | 0.098 | 0.12 |
| PT max | **14.90 [13.10, 18.50]** | **16.70 [14.10, 25.05]** | **<0.001** | **0.285** |
| ALT max | **44.00 [22.00, 105.50]** | **90.00 [37.00, 452.50]** | **<0.001** | **0.399** |
| AST max | **65.00 [32.00, 154.00]** | **153.50 [66.25, 694.50]** | **<0.001** | **0.38** |
| ABL min | **2.70 [2.27, 3.20]** | **2.60 [2.20, 3.00]** | **<0.01** | **0.179** |
| ABL max | 3.10 [2.70, 3.50] | 3.00 [2.70, 3.50] | 0.077 | 0.074 |
| Ca min | **7.60 [7.10, 8.10]** | **7.40 [6.90, 7.90]** | **<0.001** | **0.2** |
| Ca max | 8.80 [8.30, 9.30] | 8.80 [8.30, 9.50] | 0.057 | 0.12 |
| Na min | 136.00 [132.00, 138.00] | 135.00 [132.00, 138.00] | 0.649 | 0.02 |
| Na max | **143.00 [140.00, 147.00]** | **144.00 [141.00, 149.00]** | **<0.001** | **0.171** |
| K min | 3.40 [3.10, 3.70] | 3.40 [3.10, 3.70] | 0.424 | 0.015 |
| K max | **4.60 [4.20, 5.20]** | **5.00 [4.50, 5.77]** | **<0.001** | **0.315** |
| CRP max | 152.60 [65.95, 251.85] | 175.50 [89.55, 252.55] | 0.491 | 0.123 |

Supplementary Table 2. Baseline characteristics after propensity score matching of cohort

|  | **non-SIMI (N=408)** | **SIMI (N=408)** | **p-value** | **SMD** |
| --- | --- | --- | --- | --- |
| Age | 61.00 [50.00, 74.00] | 63.00 [51.00, 75.00] | 0.294 | 0.049 |
| Gender (Female) | **847 (45.44%)** | **232 (40.63%)** | **<0.05** | **0.097** |
| Mechanical ventilation (YES) | **1024 (54.94%)** | **415 (72.68%)** | **<0.001** | **0.376** |
| Continuous Renal Replacement Therapy (YES) | **69 (3.70%)** | **50 (8.76%)** | **<0.001** | **0.21** |
| Sedative Use (YES) | **974 (52.25%)** | **351 (61.47%)** | **<0.001** | **0.187** |
| Albumin Use (YES) | 205 (11.00%) | 70 (12.26%) | 0.449 | 0.039 |
| Vasopressor (YES) | **760 (40.77%)** | **345 (60.42%)** | **<0.001** | **0.401** |
| Hypertension (YES) | 980 (52.58%) | 293 (51.31%) | 0.631 | 0.025 |
| Diabetes (YES) | 430 (23.07%) | 129 (22.59%) | 0.857 | 0.011 |
| Renal Disease (YES) | 215 (11.53%) | 77 (13.49%) | 0.237 | 0.059 |
| Liver Disease (YES) | 260 (13.95%) | 64 (11.21%) | 0.106 | 0.083 |
| COPD (YES) | 221 (11.86%) | 64 (11.21%) | 0.729 | 0.02 |
| Septic Shock (YES) | **487 (26.13%)** | **193 (33.80%)** | **<0.001** | **0.168** |
| MAP min | **58.00 [50.00, 65.00]** | **55.00 [47.00, 63.00]** | **<0.001** | **0.214** |
| MAP max | 103.00 [92.00, 118.00] | 105.00 [93.00, 120.00] | 0.085 | 0.124 |
| Heart Rate min | 72.00 [61.00, 84.00] | 75.00 [61.00, 87.00] | 0.112 | 0.068 |
| Heart Rate max | 108.00 [95.00, 124.00] | 110.00 [96.00, 124.00] | 0.331 | 0.031 |
| Temperature min | **36.50 [36.11, 36.78]** | **36.33 [35.44, 36.67]** | **<0.001** | **0.368** |
| Temperature max | **37.44 [37.06, 38.11]** | **37.33 [36.89, 38.00]** | **<0.001** | **0.248** |
| PH min | **7.30 [7.22, 7.37]** | **7.22 [7.13, 7.31]** | **<0.001** | **0.566** |
| PH max | 7.44 [7.39, 7.49] | 7.44 [7.38, 7.50] | 0.66 | 0.098 |
| PO2 min | **70.00 [54.00, 92.00]** | **62.00 [47.00, 80.50]** | **<0.001** | **0.257** |
| PO2 max | **153.00 [102.00, 232.00]** | **181.00 [119.50, 266.00]** | **<0.001** | **0.229** |
| PCO2 min | **35.00 [30.00, 40.00]** | **32.00 [27.00, 38.00]** | **<0.001** | **0.305** |
| PCO2 max | **47.00 [39.00, 59.00]** | **49.00 [40.00, 62.00]** | **<0.05** | **0.099** |
| HCO3 min | **20.00 [17.00, 23.00]** | **17.00 [13.00, 20.00]** | **<0.001** | **0.567** |
| HCO3 max | **27.00 [24.00, 31.00]** | **26.00 [22.00, 29.00]** | **<0.001** | **0.236** |
| Lactate min | **1.10 [0.80, 1.50]** | **1.20 [0.90, 1.90]** | **<0.001** | **0.336** |
| Lactate max | **2.20 [1.40, 3.60]** | **3.80 [2.00, 7.70]** | **<0.001** | **0.589** |
| TNT max | **0.01 [0.01, 0.03]** | **0.25 [0.15, 0.62]** | **<0.001** | **0.532** |
| BNP max | **1174.00 [240.00, 3288.00]** | **1658.00 [438.00, 7052.00]** | **<0.001** | **0.319** |
| Creatinine max | **1.20 [0.80, 1.90]** | **2.00 [1.20, 3.60]** | **<0.001** | **0.501** |
| BUN min | **13.00 [9.00, 22.00]** | **19.00 [12.00, 30.00]** | **<0.001** | **0.352** |
| BUN max | **27.00 [17.00, 46.00]** | **39.00 [25.00, 68.00]** | **<0.001** | **0.435** |
| WBC min | 7.80 [5.50, 10.80] | 8.20 [5.45, 11.80] | 0.09 | 0.134 |
| WBC max | **15.30 [10.80, 21.30]** | **18.80 [12.90, 26.20]** | **<0.001** | **0.294** |
| HB min | **8.90 [7.40, 10.50]** | **8.30 [6.90, 10.20]** | **<0.001** | **0.133** |
| HB max | **11.40 [10.10, 13.00]** | **11.70 [10.20, 13.40]** | **<0.05** | **0.144** |
| PLT min | **145.50 [91.00, 206.00]** | **116.00 [69.50, 169.50]** | **<0.001** | **0.346** |
| PLT max | **243.00 [163.00, 356.00]** | **221.00 [148.50, 318.50]** | **<0.01** | **0.153** |
| INR min | **1.20 [1.10, 1.30]** | **1.20 [1.10, 1.40]** | **<0.05** | **0.13** |
| INR max | **1.40 [1.20, 1.70]** | **1.50 [1.30, 2.30]** | **<0.001** | **0.275** |
| DDimer min | **1680.00 [1042.50, 4621.00]** | **3169.00 [1286.00, 7012.00]** | **<0.001** | **0.357** |
| DDimer max | **4273.00 [1769.00, 8389.00]** | **6765.00 [2321.00, 13978.50]** | **<0.001** | **0.269** |
| FIB min | **357.00 [203.00, 547.75]** | **284.00 [150.50, 474.00]** | **<0.001** | **0.26** |
| FIB max | **434.00 [252.00, 674.25]** | **392.00 [226.00, 630.50]** | **<0.01** | **0.153** |
| PT min | 12.80 [11.60, 14.40] | 12.90 [11.60, 15.35] | 0.175 | 0.119 |
| PT max | **14.80 [13.10, 18.10]** | **16.50 [14.00, 23.90]** | **<0.001** | **0.288** |
| ALT max | **44.00 [22.00, 104.00]** | **85.00 [34.50, 368.00]** | **<0.001** | **0.384** |
| AST max | **65.00 [32.00, 150.00]** | **134.00 [60.50, 603.50]** | **<0.001** | **0.363** |
| ABL min | **2.80 [2.30, 3.30]** | **2.70 [2.25, 3.10]** | **<0.001** | **0.201** |
| ABL max | 3.10 [2.70, 3.50] | 3.00 [2.70, 3.50] | 0.059 | 0.076 |
| Ca min | **7.60 [7.10, 8.10]** | **7.40 [6.90, 7.90]** | **<0.001** | **0.19** |
| Ca max | 8.80 [8.30, 9.30] | 8.80 [8.30, 9.50] | 0.069 | 0.116 |
| Na min | 136.00 [132.00, 138.00] | 135.00 [132.00, 138.00] | 0.663 | 0.019 |
| Na max | **143.00 [140.00, 147.00]** | **144.00 [141.00, 149.00]** | **<0.001** | **0.17** |
| K min | 3.40 [3.10, 3.70] | 3.40 [3.10, 3.70] | 0.419 | 0.015 |
| K max | **4.60 [4.20, 5.20]** | **5.00 [4.50, 5.80]** | **<0.001** | **0.316** |
| CRP max | 99.70 [38.60, 204.40] | 99.30 [38.60, 217.50] | 0.712 | 0.023 |

Supplementary Table 3. Baseline characteristics of the machine learning cohort

|  | **non-SIMI (N=1864)** | **SIMI (N=571)** | **p-value** | **SMD** |
| --- | --- | --- | --- | --- |
| Age | 61.00 [50.00, 74.00] | 63.00 [51.00, 75.00] | 0.294 | 0.049 |
| Gender (Female) | **847 (45.44%)** | **232 (40.63%)** | **<0.05** | **0.097** |
| Mechanical ventilation (YES) | **1024 (54.94%)** | **415 (72.68%)** | **<0.001** | **0.376** |
| Continuous Renal Replacement Therapy (YES) | **69 (3.70%)** | **50 (8.76%)** | **<0.001** | **0.21** |
| Sedative Use (YES) | **974 (52.25%)** | **351 (61.47%)** | **<0.001** | **0.187** |
| Albumin Use (YES) | 205 (11.00%) | 70 (12.26%) | 0.449 | 0.039 |
| Vasopressor (YES) | **760 (40.77%)** | **345 (60.42%)** | **<0.001** | **0.401** |
| Hypertension (YES) | 980 (52.58%) | 293 (51.31%) | 0.631 | 0.025 |
| Diabetes (YES) | 430 (23.07%) | 129 (22.59%) | 0.857 | 0.011 |
| Renal Disease (YES) | 215 (11.53%) | 77 (13.49%) | 0.237 | 0.059 |
| Liver Disease (YES) | 260 (13.95%) | 64 (11.21%) | 0.106 | 0.083 |
| COPD (YES) | 221 (11.86%) | 64 (11.21%) | 0.729 | 0.02 |
| Septic Shock (YES) | **487 (26.13%)** | **193 (33.80%)** | **<0.001** | **0.168** |
| MAP first | 82.00 [70.00, 95.00] | 83.00 [71.00, 94.00] | 0.832 | 0.026 |
| Heart Rate first | **93.00 [78.00, 107.00]** | **96.00 [81.00, 111.50]** | **<0.05** | **0.103** |
| Temperature first | **36.83 [36.44, 37.28]** | **36.67 [36.06, 37.17]** | **<0.001** | **0.314** |
| PH first | **7.35 [7.28, 7.41]** | **7.29 [7.19, 7.37]** | **<0.001** | **0.458** |
| PO2 first | 101.00 [73.00, 161.75] | 102.00 [67.75, 174.25] | 0.946 | 0.065 |
| PCO2 first | **41.00 [35.00, 49.00]** | **39.00 [33.00, 50.00]** | **<0.05** | **0.095** |
| HCO3 first | **22.00 [19.00, 25.00]** | **19.00 [16.00, 23.00]** | **<0.001** | **0.442** |
| Lactate first | **1.90 [1.20, 3.00]** | **3.00 [1.60, 5.53]** | **<0.001** | **0.567** |
| TNT first | **0.01 [0.01, 0.03]** | **0.19 [0.11, 0.44]** | **<0.001** | **0.502** |
| BNP first | **996.50 [237.50, 3068.25]** | **1692.00 [758.50, 5805.50]** | **<0.01** | **0.288** |
| Creatinine first | **1.00 [0.70, 1.50]** | **1.40 [1.00, 2.20]** | **<0.001** | **0.361** |
| BUN first | **19.00 [13.00, 32.00]** | **26.00 [17.00, 44.00]** | **<0.001** | **0.344** |
| WBC first | **11.60 [7.80, 16.60]** | **13.50 [9.20, 19.50]** | **<0.001** | **0.239** |
| HB first | 11.00 [9.40, 12.50] | 11.10 [9.20, 13.00] | 0.222 | 0.079 |
| PLT first | **193.00 [132.00, 262.00]** | **185.00 [125.00, 254.00]** | **<0.05** | **0.094** |
| INR first | **1.20 [1.10, 1.50]** | **1.30 [1.10, 1.80]** | **<0.001** | **0.179** |
| DDimer first | **2280.00 [1152.50, 5691.00]** | **4951.00 [2450.00, 8702.00]** | **<0.001** | **0.571** |
| FIB first | **341.00 [187.25, 565.75]** | **266.00 [138.50, 446.50]** | **<0.001** | **0.323** |
| PT first | **13.80 [12.40, 16.30]** | **14.50 [12.70, 19.00]** | **<0.001** | **0.174** |
| ALT first | **33.00 [18.00, 69.00]** | **63.00 [28.00, 215.25]** | **<0.001** | **0.333** |
| AST first | **47.00 [26.00, 97.00]** | **104.50 [49.00, 362.00]** | **<0.001** | **0.326** |
| ABL first | 2.90 [2.50, 3.40] | 2.90 [2.40, 3.30] | 0.051 | 0.116 |
| Ca first | **8.10 [7.50, 8.70]** | **8.00 [7.40, 8.60]** | **<0.05** | **0.056** |
| Na first | **139.00 [135.00, 142.00]** | **139.00 [136.00, 142.00]** | **<0.01** | **0.105** |
| K first | **4.10 [3.70, 4.60]** | **4.20 [3.70, 4.90]** | **<0.001** | **0.19** |
| CRP first | 129.80 [58.95, 218.15] | 120.10 [66.00, 230.35] | 0.913 | 0.026 |
| CKMB first | **4.00 [2.00, 7.25]** | **13.50 [6.00, 31.00]** | **<0.001** | **0.545** |

Supplementary Table 4. Unadjusted log-rank test for outcomes of original cohort

| Group | Outcome | HR^1^ | 95% CI^1^ | p-value |
| --- | --- | --- | --- | --- |
| non-SIMI |  | — | — | — |
| SIMI | hos-die mortality | 2.51 | 2.04, 3.09 | <0.001 |
| SIMI | 28-day mortality | 2.60 | 2.15, 3.16 | <0.001 |
| SIMI | 180-day mortality | 2.39 | 2.01, 2.84 | <0.001 |
| SIMI | 1-year mortality | 2.24 | 1.89, 2.64 | <0.001 |
|  | ^1^HR = Hazard Ratio, CI = Confidence Interval; | | | |

Supplementary Table 5. Multivariate logistic model adjusted with Boruta selected covariate (Variables other than the group listed in the table) for hos-die mortality of original cohort

| **Characteristic** | **OR**^1^ | **95% CI**^1^ | **p-value** |
| --- | --- | --- | --- |
| Group |  |  |  |
| non-SIMI | 1.00 | Reference |  |
| SIMI | 1.55 | 1.15, 2.09 | 0.004 |
| K_max | 1.05 | 0.92, 1.21 | 0.465 |
| K_min | 1.18 | 0.86, 1.63 | 0.297 |
| Na_max | 1.01 | 0.99, 1.03 | 0.241 |
| Na_min | 0.99 | 0.96, 1.01 | 0.267 |
| Ca_max | 1.05 | 0.93, 1.19 | 0.438 |
| ABL_max | 1.00 | 0.75, 1.35 | 0.982 |
| ABL_min | 0.93 | 0.67, 1.28 | 0.641 |
| AST_max | 1.00 | 1.00, 1.00 | 0.549 |
| ALT_max | 1.00 | 1.00, 1.00 | 0.141 |
| PT_max | 1.00 | 0.97, 1.03 | 0.876 |
| PT_min | 1.02 | 0.94, 1.11 | 0.649 |
| FIB_max | 1.00 | 1.00, 1.00 | 0.575 |
| FIB_min | 1.00 | 1.00, 1.00 | 0.24 |
| DDimer_max | 1.00 | 1.00, 1.00 | 0.313 |
| DDimer_min | 1.00 | 1.00, 1.00 | 0.457 |
| INR_max | 1.11 | 0.88, 1.61 | 0.455 |
| INR_min | 1.12 | 0.47, 2.72 | 0.791 |
| PLT_max | 1.00 | 0.99, 1.00 | <0.001 |
| PLT_min | 1.00 | 1.00, 1.01 | 0.016 |
| HB_max | 1.10 | 1.01, 1.21 | 0.038 |
| HB_min | 0.95 | 0.85, 1.05 | 0.319 |
| WBC_max | 1.01 | 1.00, 1.03 | 0.126 |
| WBC_min | 1.02 | 0.99, 1.06 | 0.185 |
| BUN_max | 1.01 | 1.00, 1.01 | 0.077 |
| BUN_min | 1.00 | 0.99, 1.01 | 0.719 |
| Creatinine_max | 0.96 | 0.87, 1.06 | 0.397 |
| BNP_max | 1.00 | 1.00, 1.00 | 0.973 |
| Lactate_max | 1.15 | 1.08, 1.22 | <0.001 |
| Lactate_min | 1.54 | 1.26, 1.91 | <0.001 |
| HCO3_max | 0.90 | 0.86, 0.94 | <0.001 |
| HCO3_min | 1.02 | 0.97, 1.07 | 0.442 |
| PCO2_max | 1.04 | 1.03, 1.05 | <0.001 |
| PCO2_min | 0.99 | 0.96, 1.01 | 0.217 |
| PO2_max | 1.00 | 1.00, 1.00 | 0.006 |
| PO2_min | 1.00 | 0.99, 1.00 | 0.024 |
| PH_max | 3.88 | 0.25, 60.1 | 0.331 |
| PH_min | 0.35 | 0.04, 3.15 | 0.346 |
| Temperature_max | 0.89 | 0.76, 1.04 | 0.136 |
| Temperature_min | 0.84 | 0.73, 0.97 | 0.015 |
| Heart_Rate_max | 1.00 | 0.99, 1.00 | 0.263 |
| MAP_min | 1.00 | 0.99, 1.01 | 0.745 |
| Vasopressor |  |  |  |
| YES | 1.00 | Reference |  |
| NO | 0.73 | 0.54, 0.99 | 0.042 |
| Continuous_Renal_Replacement_Therapy |  |  |  |
| YES | 1.00 | Reference |  |
| NO | 1.09 | 0.59, 2.04 | 0.775 |
| Mechanical_ventilation |  |  |  |
| YES | 1.00 | Reference |  |
| NO | 0.35 | 0.24, 0.50 | <0.001 |
| Age | 1.01 | 1.00, 1.02 | 0.036 |
| ^1^OR = Odds Ratio, CI = Confidence Interval | | | |

Supplementary Table 6. Multivariate logistic model adjusted with unbalanced covariates (Variables other than the group listed in the table) selected for hos-die mortality of original cohort

| **Characteristic** | **OR**^1^ | **95% CI**^1^ | **p-value** |
| --- | --- | --- | --- |
| Group |  |  |  |
| non-SIMI | 1.00 | Reference |  |
| SIMI | 1.52 | 1.13, 2.03 | 0.005 |
| Mechanical_ventilation |  |  |  |
| YES | 1.00 | Reference |  |
| NO | 0.30 | 0.20, 0.46 | <0.001 |
| Continuous_Renal_Replacement_Therapy |  |  |  |
| YES | 1.00 | Reference |  |
| NO | 0.99 | 0.54, 1.82 | 0.976 |
| Sedative_Use |  |  |  |
| YES | 1.00 | Reference |  |
| NO | 1.43 | 0.96, 2.14 | 0.082 |
| Vasopressor |  |  |  |
| YES | 1.00 | Reference |  |
| NO | 0.68 | 0.49, 0.95 | 0.023 |
| Septic_Shock |  |  |  |
| YES | 1.00 | Reference |  |
| NO | 1.02 | 0.74, 1.42 | 0.884 |
| MAP_min | 1.00 | 0.99, 1.01 | 0.96 |
| MAP_max | 1.00 | 1.00, 1.01 | 0.382 |
| Temperature_min | 0.88 | 0.76, 1.02 | 0.075 |
| Temperature_max | 0.85 | 0.73, 0.99 | 0.041 |
| PH_min | 0.00 | 0.00, 0.02 | <0.001 |
| PO2_min | 0.99 | 0.99, 1.00 | 0.001 |
| PO2_max | 1.00 | 1.00, 1.00 | <0.001 |
| PCO2_min | 0.99 | 0.97, 1.01 | 0.499 |
| HCO3_min | 1.06 | 1.01, 1.12 | 0.015 |
| HCO3_max | 0.95 | 0.92, 0.98 | 0.003 |
| Lactate_min | 1.57 | 1.29, 1.93 | <0.001 |
| Lactate_max | 1.11 | 1.05, 1.17 | <0.001 |
| BNP_max | 1.00 | 1.00, 1.00 | 0.572 |
| Creatinine_max | 0.94 | 0.85, 1.03 | 0.193 |
| BUN_min | 1.00 | 0.99, 1.02 | 0.362 |
| BUN_max | 1.01 | 1.00, 1.01 | 0.02 |
| WBC_min | 1.03 | 1.00, 1.07 | 0.054 |
| WBC_max | 1.01 | 1.00, 1.03 | 0.176 |
| HB_min | 0.94 | 0.84, 1.05 | 0.26 |
| HB_max | 1.10 | 1.01, 1.21 | 0.038 |
| PLT_min | 1.00 | 1.00, 1.01 | 0.03 |
| PLT_max | 1.00 | 0.99, 1.00 | <0.001 |
| INR_min | 1.10 | 0.45, 2.84 | 0.833 |
| INR_max | 1.23 | 0.96, 1.89 | 0.176 |
| DDimer_min | 1.00 | 1.00, 1.00 | 0.529 |
| DDimer_max | 1.00 | 1.00, 1.00 | 0.297 |
| FIB_min | 1.00 | 1.00, 1.00 | 0.22 |
| FIB_max | 1.00 | 1.00, 1.00 | 0.893 |
| PT_min | 1.03 | 0.94, 1.12 | 0.518 |
| PT_max | 0.99 | 0.95, 1.02 | 0.613 |
| ALT_max | 1.00 | 1.00, 1.00 | 0.173 |
| AST_max | 1.00 | 1.00, 1.00 | 0.557 |
| ABL_min | 0.94 | 0.72, 1.22 | 0.631 |
| Ca_min | 1.06 | 0.88, 1.28 | 0.558 |
| Ca_max | 1.05 | 0.93, 1.19 | 0.401 |
| Na_max | 1.01 | 0.99, 1.03 | 0.228 |
| K_max | 1.10 | 0.97, 1.26 | 0.13 |
| ^1^OR = Odds Ratio, CI = Confidence Interval | | | |

Supplementary Table 7. Multivariate logistic model adjusted with Boruta selected covariates (Variables other than the group listed in the table) using IPTW for hos-die mortality of cohort

| **Characteristic** | **OR**^1^ | **95% CI**^1^ | **p-value** |
| --- | --- | --- | --- |
| Group |  |  |  |
| non-SIMI | 1.00 | Reference |  |
| SIMI | 1.38 | 1.12, 1.69 | 0.002 |
| K_max | 1.06 | 0.95, 1.19 | 0.299 |
| K_min | 1.40 | 1.10, 1.78 | 0.006 |
| Na_max | 1.01 | 1.00, 1.03 | 0.072 |
| Na_min | 0.98 | 0.97, 1.00 | 0.012 |
| Ca_max | 1.04 | 0.95, 1.15 | 0.405 |
| ABL_max | 0.97 | 0.78, 1.22 | 0.822 |
| ABL_min | 0.98 | 0.77, 1.25 | 0.887 |
| AST_max | 1.00 | 1.00, 1.00 | 0.501 |
| ALT_max | 1.00 | 1.00, 1.00 | 0.057 |
| PT_max | 1.00 | 0.97, 1.01 | 0.768 |
| PT_min | 1.04 | 0.98, 1.12 | 0.206 |
| FIB_max | 1.00 | 1.00, 1.00 | 0.357 |
| FIB_min | 1.00 | 1.00, 1.00 | 0.039 |
| DDimer_max | 1.00 | 1.00, 1.00 | 0.112 |
| DDimer_min | 1.00 | 1.00, 1.00 | 0.53 |
| INR_max | 1.16 | 0.97, 1.54 | 0.175 |
| INR_min | 0.90 | 0.45, 1.77 | 0.75 |
| PLT_max | 1.0 | 0.99, 1.00 | <0.001 |
| PLT_min | 1.00 | 1.00, 1.01 | <0.001 |
| HB_max | 1.16 | 1.08, 1.24 | <0.001 |
| HB_min | 0.92 | 0.85, 1.00 | 0.043 |
| WBC_max | 1.01 | 1.00, 1.03 | 0.057 |
| WBC_min | 1.02 | 1.00, 1.05 | 0.075 |
| BUN_max | 1.01 | 1.00, 1.01 | <0.001 |
| BUN_min | 1.00 | 0.99, 1.01 | 0.91 |
| Creatinine_max | 0.94 | 0.87, 1.02 | 0.141 |
| BNP_max | 1.00 | 1.00, 1.00 | 0.632 |
| Lactate_max | 1.16 | 1.11, 1.22 | <0.001 |
| Lactate_min | 1.55 | 1.33, 1.82 | <0.001 |
| HCO3_max | 0.88 | 0.86, 0.91 | <0.001 |
| HCO3_min | 1.02 | 0.98, 1.06 | 0.284 |
| PCO2_max | 1.05 | 1.03, 1.06 | <0.001 |
| PCO2_min | 0.98 | 0.96, 1.00 | 0.015 |
| PO2_max | 1.00 | 1.00, 1.00 | <0.001 |
| PO2_min | 1.00 | 0.99, 1.00 | 0.003 |
| PH_max | 10.9 | 1.36, 88.2 | 0.025 |
| PH_min | 0.49 | 0.09, 2.63 | 0.402 |
| Temperature_max | 0.90 | 0.80, 1.02 | 0.102 |
| Temperature_min | 0.81 | 0.73, 0.91 | <0.001 |
| Heart_Rate_max | 1.00 | 0.99, 1.00 | 0.168 |
| MAP_min | 1.00 | 0.99, 1.01 | 0.754 |
| Vasopressor |  |  |  |
| YES | 1.00 | Reference |  |
| NO | 0.69 | 0.55, 0.86 | 0.001 |
| Continuous_Renal_Replacement_Therapy |  |  |  |
| YES | 1.00 | Reference |  |
| NO | 0.97 | 0.61, 1.55 | 0.906 |
| Mechanical_ventilation |  |  |  |
| YES | 1.00 | Reference |  |
| NO | 0.39 | 0.30, 0.51 | <0.001 |
| Age | 1.01 | 1.01, 1.02 | <0.001 |
| ^1^OR = Odds Ratio, CI = Confidence Interval | | | |

Supplementary Table 8. Multivariate logistic model adjusted with unbalanced covariates (Variables other than the group listed in the table) using IPTW for hos-die mortality of cohort

| **Characteristic** | **OR**^1^ | **95% CI**^1^ | **p-value** |
| --- | --- | --- | --- |
| Group |  |  |  |
| non-SIMI | 1.00 | Reference |  |
| SIMI | 1.32 | 1.08, 1.61 | 0.006 |
| Mechanical_ventilation |  |  |  |
| YES | 1.00 | Reference |  |
| NO | 0.36 | 0.26, 0.50 | <0.001 |
| Continuous_Renal_Replacement_Therapy |  |  |  |
| YES | 1.00 | Reference |  |
| NO | 0.88 | 0.56, 1.38 | 0.575 |
| Sedative_Use |  |  |  |
| YES | 1.00 | Reference |  |
| NO | 1.37 | 1.02, 1.86 | 0.039 |
| Vasopressor |  |  |  |
| YES | 1.00 | Reference |  |
| NO | 0.64 | 0.50, 0.82 | <0.001 |
| Septic_Shock |  |  |  |
| YES | 1.00 | Reference |  |
| NO | 1.09 | 0.86, 1.40 | 0.472 |
| MAP_min | 1.00 | 0.99, 1.01 | 0.811 |
| MAP_max | 1.00 | 1.00, 1.01 | 0.218 |
| Temperature_min | 0.86 | 0.78, 0.96 | 0.008 |
| Temperature_max | 0.86 | 0.77, 0.97 | 0.013 |
| PH_min | 0.01 | 0.00, 0.02 | <0.001 |
| PO2_min | 0.99 | 0.99, 1.00 | <0.001 |
| PO2_max | 1.00 | 1.00, 1.00 | <0.001 |
| PCO2_min | 0.98 | 0.97, 1.00 | 0.047 |
| HCO3_min | 1.07 | 1.03, 1.12 | <0.001 |
| HCO3_max | 0.94 | 0.91, 0.96 | <0.001 |
| Lactate_min | 1.57 | 1.35, 1.84 | <0.001 |
| Lactate_max | 1.13 | 1.09, 1.18 | <0.001 |
| BNP_max | 1.00 | 1.00, 1.00 | 0.607 |
| Creatinine_max | 0.92 | 0.85, 0.99 | 0.025 |
| BUN_min | 1.00 | 1.00, 1.01 | 0.242 |
| BUN_max | 1.01 | 1.01, 1.02 | <0.001 |
| WBC_min | 1.03 | 1.01, 1.06 | 0.012 |
| WBC_max | 1.01 | 1.00, 1.02 | 0.066 |
| HB_min | 0.92 | 0.85, 1.00 | 0.041 |
| HB_max | 1.14 | 1.07, 1.22 | <0.001 |
| PLT_min | 1.00 | 1.00, 1.01 | <0.001 |
| PLT_max | 1.0 | 0.99, 1.00 | <0.001 |
| INR_min | 0.84 | 0.42, 1.73 | 0.632 |
| INR_max | 1.29 | 1.07, 1.81 | 0.035 |
| DDimer_min | 1.00 | 1.00, 1.00 | 0.165 |
| DDimer_max | 1.00 | 1.00, 1.00 | 0.109 |
| FIB_min | 1.00 | 1.00, 1.00 | 0.052 |
| FIB_max | 1.00 | 1.00, 1.00 | 0.972 |
| PT_min | 1.06 | 0.99, 1.14 | 0.103 |
| PT_max | 0.99 | 0.95, 1.01 | 0.266 |
| ALT_max | 1.00 | 1.00, 1.00 | 0.066 |
| AST_max | 1.00 | 1.00, 1.00 | 0.523 |
| ABL_min | 1.00 | 0.83, 1.22 | 0.973 |
| Ca_min | 1.01 | 0.87, 1.17 | 0.909 |
| Ca_max | 1.06 | 0.96, 1.16 | 0.24 |
| Na_max | 1.01 | 1.00, 1.02 | 0.18 |
| K_max | 1.13 | 1.02, 1.26 | 0.021 |
| ^1^OR = Odds Ratio, CI = Confidence Interval | | | |

Supplementary Table 9. Survey-weighted generalized linear model adjusted with Boruta selected covariates (Variables other than the group listed in the table) using IPTW for hos-die mortality of cohort

| **Characteristic** | **OR**^1^ | **95% CI**^1^ | **p-value** |
| --- | --- | --- | --- |
| Group |  |  |  |
| non-SIMI | 1.00 | Reference |  |
| SIMI | 1.38 | 1.02, 1.86 | 0.038 |
| K_max | 1.06 | 0.91, 1.23 | 0.454 |
| K_min | 1.40 | 0.99, 1.99 | 0.06 |
| Na_max | 1.01 | 0.99, 1.04 | 0.312 |
| Na_min | 0.98 | 0.96, 1.00 | 0.077 |
| Ca_max | 1.04 | 0.93, 1.16 | 0.477 |
| ABL_max | 0.97 | 0.71, 1.33 | 0.871 |
| ABL_min | 0.98 | 0.69, 1.41 | 0.924 |
| AST_max | 1.00 | 1.00, 1.00 | 0.576 |
| ALT_max | 1.00 | 1.00, 1.00 | 0.107 |
| PT_max | 1.00 | 0.98, 1.01 | 0.717 |
| PT_min | 1.04 | 0.97, 1.13 | 0.274 |
| FIB_max | 1.00 | 1.00, 1.00 | 0.517 |
| FIB_min | 1.00 | 1.00, 1.00 | 0.162 |
| DDimer_max | 1.00 | 1.00, 1.00 | 0.242 |
| DDimer_min | 1.00 | 1.00, 1.00 | 0.635 |
| INR_max | 1.16 | 0.97, 1.37 | 0.095 |
| INR_min | 0.90 | 0.43, 1.84 | 0.765 |
| PLT_max | 1.0 | 0.99, 1.00 | <0.001 |
| PLT_min | 1.00 | 1.00, 1.01 | 0.008 |
| HB_max | 1.16 | 1.04, 1.29 | 0.006 |
| HB_min | 0.92 | 0.81, 1.04 | 0.178 |
| WBC_max | 1.01 | 1.00, 1.03 | 0.148 |
| WBC_min | 1.02 | 0.99, 1.06 | 0.223 |
| BUN_max | 1.01 | 1.00, 1.02 | 0.021 |
| BUN_min | 1.00 | 0.99, 1.01 | 0.939 |
| Creatinine_max | 0.94 | 0.86, 1.04 | 0.243 |
| BNP_max | 1.00 | 1.00, 1.00 | 0.734 |
| Lactate_max | 1.16 | 1.09, 1.24 | <0.001 |
| Lactate_min | 1.55 | 1.17, 2.05 | 0.002 |
| HCO3_max | 0.88 | 0.84, 0.93 | <0.001 |
| HCO3_min | 1.02 | 0.96, 1.08 | 0.495 |
| PCO2_max | 1.05 | 1.03, 1.06 | <0.001 |
| PCO2_min | 0.98 | 0.95, 1.00 | 0.096 |
| PO2_max | 1.00 | 1.00, 1.00 | 0.003 |
| PO2_min | 1.00 | 0.99, 1.00 | 0.048 |
| PH_max | 10.9 | 0.37, 319 | 0.167 |
| PH_min | 0.49 | 0.04, 5.56 | 0.562 |
| Temperature_max | 0.90 | 0.75, 1.10 | 0.304 |
| Temperature_min | 0.81 | 0.69, 0.96 | 0.015 |
| Heart_Rate_max | 1.00 | 0.99, 1.00 | 0.388 |
| MAP_min | 1.00 | 0.99, 1.01 | 0.837 |
| Vasopressor |  |  |  |
| YES | 1.00 | Reference |  |
| NO | 0.69 | 0.48, 0.98 | 0.036 |
| Continuous_Renal_Replacement_Therapy |  |  |  |
| YES | 1.00 | Reference |  |
| NO | 0.97 | 0.48, 1.99 | 0.938 |
| Mechanical_ventilation |  |  |  |
| YES | 1.00 | Reference |  |
| NO | 0.39 | 0.25, 0.60 | <0.001 |
| Age | 1.01 | 1.00, 1.02 | 0.009 |
| ^1^OR = Odds Ratio, CI = Confidence Interval | | | |

Supplementary Table 10. Multivariate logistic model adjusted with Boruta selected covariates (Variables other than the group listed in the table) for 28-day mortality of original cohort

| **Characteristic** | **OR**^1^ | **95% CI**^1^ | **p-value** |
| --- | --- | --- | --- |
| Group |  |  |  |
| non-SIMI | 1.00 | Reference |  |
| SIMI | 1.48 | 1.14, 1.93 | 0.004 |
| K_max | 1.11 | 0.98, 1.27 | 0.102 |
| K_min | 1.09 | 0.82, 1.45 | 0.544 |
| Na_max | 1.04 | 1.02, 1.06 | <0.001 |
| Na_min | 0.99 | 0.97, 1.01 | 0.423 |
| Ca_max | 1.02 | 0.91, 1.14 | 0.7 |
| ABL_max | 0.89 | 0.69, 1.16 | 0.411 |
| ABL_min | 0.88 | 0.66, 1.17 | 0.364 |
| AST_max | 1.00 | 1.00, 1.00 | 0.594 |
| ALT_max | 1.00 | 1.00, 1.00 | 0.405 |
| PT_max | 0.99 | 0.95, 1.02 | 0.735 |
| PT_min | 1.10 | 1.02, 1.19 | 0.02 |
| FIB_max | 1.00 | 1.00, 1.00 | 0.092 |
| FIB_min | 1.00 | 1.00, 1.00 | 0.204 |
| DDimer_max | 1.00 | 1.00, 1.00 | 0.57 |
| DDimer_min | 1.00 | 1.00, 1.00 | 0.627 |
| INR_max | 1.17 | 0.91, 1.89 | 0.338 |
| INR_min | 0.86 | 0.38, 1.88 | 0.716 |
| PLT_max | 1.00 | 1.00, 1.00 | <0.001 |
| PLT_min | 1.00 | 1.00, 1.00 | 0.058 |
| HB_max | 1.07 | 0.98, 1.16 | 0.137 |
| HB_min | 0.97 | 0.88, 1.07 | 0.578 |
| WBC_max | 1.00 | 0.99, 1.02 | 0.909 |
| WBC_min | 1.04 | 1.00, 1.07 | 0.034 |
| BUN_max | 1.00 | 1.00, 1.01 | 0.274 |
| BUN_min | 1.01 | 1.00, 1.02 | 0.049 |
| Creatinine_max | 0.95 | 0.86, 1.04 | 0.27 |
| BNP_max | 1.00 | 1.00, 1.00 | 0.647 |
| Lactate_max | 1.10 | 1.04, 1.16 | <0.001 |
| Lactate_min | 1.50 | 1.24, 1.83 | <0.001 |
| HCO3_max | 0.90 | 0.86, 0.93 | <0.001 |
| HCO3_min | 1.02 | 0.97, 1.06 | 0.509 |
| PCO2_max | 1.04 | 1.03, 1.06 | <0.001 |
| PCO2_min | 0.98 | 0.96, 1.00 | 0.119 |
| PO2_max | 1.00 | 1.00, 1.00 | 0.016 |
| PO2_min | 1.00 | 0.99, 1.00 | 0.019 |
| PH_max | 4.27 | 0.39, 47.3 | 0.236 |
| PH_min | 2.25 | 0.31, 16.5 | 0.424 |
| Temperature_max | 0.84 | 0.73, 0.97 | 0.021 |
| Temperature_min | 0.82 | 0.72, 0.93 | 0.002 |
| Heart_Rate_max | 1.00 | 1.00, 1.01 | 0.742 |
| MAP_min | 1.00 | 0.99, 1.01 | 0.549 |
| Vasopressor |  |  |  |
| YES | 1.00 | Reference |  |
| NO | 0.86 | 0.66, 1.12 | 0.256 |
| Continuous_Renal_Replacement_Therapy |  |  |  |
| YES | 1.00 | Reference |  |
| NO | 1.30 | 0.72, 2.39 | 0.389 |
| Mechanical_ventilation |  |  |  |
| YES | 1.00 | Reference |  |
| NO | 0.38 | 0.28, 0.52 | <0.001 |
| Age | 1.02 | 1.01, 1.03 | <0.001 |
| ^1^OR = Odds Ratio, CI = Confidence Interval | | | |

Supplementary Table 11. Multivariate logistic model adjusted with unbalanced covariates (Variables other than the group listed in the table) selected for 28-day mortality of original cohort

| **Characteristic** | **OR**^1^ | **95% CI**^1^ | **p-value** |
| --- | --- | --- | --- |
| Group |  |  |  |
| non-SIMI | 1.00 | Reference |  |
| SIMI | 1.45 | 1.11, 1.88 | 0.006 |
| Mechanical_ventilation |  |  |  |
| YES | 1.00 | Reference |  |
| NO | 0.35 | 0.24, 0.51 | <0.001 |
| Continuous_Renal_Replacement_Therapy |  |  |  |
| YES | 1.00 | Reference |  |
| NO | 1.24 | 0.69, 2.25 | 0.471 |
| Sedative_Use |  |  |  |
| YES | 1.00 | Reference |  |
| NO | 1.45 | 1.02, 2.07 | 0.041 |
| Vasopressor |  |  |  |
| YES | 1.00 | Reference |  |
| NO | 0.76 | 0.58, 1.01 | 0.057 |
| Septic_Shock |  |  |  |
| YES | 1.00 | Reference |  |
| NO | 1.08 | 0.81, 1.43 | 0.616 |
| MAP_min | 0.99 | 0.99, 1.00 | 0.162 |
| MAP_max | 1.00 | 1.00, 1.01 | 0.386 |
| Temperature_min | 0.86 | 0.76, 0.98 | 0.022 |
| Temperature_max | 0.82 | 0.71, 0.93 | 0.004 |
| PH_min | 0.03 | 0.01, 0.14 | <0.001 |
| PO2_min | 1.0 | 0.99, 1.00 | <0.001 |
| PO2_max | 1.00 | 1.00, 1.00 | 0.001 |
| PCO2_min | 0.99 | 0.97, 1.01 | 0.313 |
| HCO3_min | 1.06 | 1.01, 1.11 | 0.009 |
| HCO3_max | 0.95 | 0.92, 0.98 | <0.001 |
| Lactate_min | 1.53 | 1.27, 1.85 | <0.001 |
| Lactate_max | 1.08 | 1.02, 1.14 | 0.004 |
| BNP_max | 1.00 | 1.00, 1.00 | 0.241 |
| Creatinine_max | 0.90 | 0.82, 0.99 | 0.026 |
| BUN_min | 1.02 | 1.01, 1.03 | 0.002 |
| BUN_max | 1.01 | 1.00, 1.01 | 0.032 |
| WBC_min | 1.04 | 1.01, 1.08 | 0.006 |
| WBC_max | 1.00 | 0.98, 1.01 | 0.927 |
| HB_min | 0.98 | 0.89, 1.08 | 0.693 |
| HB_max | 1.05 | 0.97, 1.14 | 0.242 |
| PLT_min | 1.00 | 1.00, 1.00 | 0.08 |
| PLT_max | 1.00 | 1.00, 1.00 | <0.001 |
| INR_min | 0.75 | 0.32, 1.65 | 0.481 |
| INR_max | 1.36 | 1.01, 2.44 | 0.16 |
| DDimer_min | 1.00 | 1.00, 1.00 | 0.033 |
| DDimer_max | 1.00 | 1.00, 1.00 | 0.568 |
| FIB_min | 1.00 | 1.00, 1.00 | 0.155 |
| FIB_max | 1.00 | 1.00, 1.00 | 0.253 |
| PT_min | 1.11 | 1.03, 1.21 | 0.008 |
| PT_max | 0.98 | 0.92, 1.01 | 0.371 |
| ALT_max | 1.00 | 1.00, 1.00 | 0.36 |
| AST_max | 1.00 | 1.00, 1.00 | 0.526 |
| ABL_min | 0.80 | 0.64, 1.00 | 0.048 |
| Ca_min | 1.03 | 0.87, 1.22 | 0.749 |
| Ca_max | 1.01 | 0.90, 1.13 | 0.829 |
| Na_max | 1.03 | 1.02, 1.05 | <0.001 |
| K_max | 1.15 | 1.02, 1.30 | 0.023 |
| ^1^OR = Odds Ratio, CI = Confidence Interval | | | |

Supplementary Table 12. Multivariate logistic model adjusted with Boruta selected covariates (Variables other than the group listed in the table) using IPTW for 28-day mortality of cohort

| **Characteristic** | **OR**^1^ | **95% CI**^1^ | **p-value** |
| --- | --- | --- | --- |
| Group |  |  |  |
| non-SIMI | 1.00 | Reference |  |
| SIMI | 1.35 | 1.14, 1.61 | <0.001 |
| K_max | 1.08 | 0.98, 1.20 | 0.142 |
| K_min | 1.40 | 1.14, 1.73 | 0.002 |
| Na_max | 1.04 | 1.02, 1.05 | <0.001 |
| Na_min | 0.99 | 0.98, 1.00 | 0.093 |
| Ca_max | 1.01 | 0.93, 1.10 | 0.768 |
| ABL_max | 0.76 | 0.62, 0.93 | 0.007 |
| ABL_min | 0.94 | 0.76, 1.16 | 0.554 |
| AST_max | 1.00 | 1.00, 1.00 | 0.401 |
| ALT_max | 1.00 | 1.00, 1.00 | 0.434 |
| PT_max | 0.98 | 0.94, 1.01 | 0.35 |
| PT_min | 1.13 | 1.06, 1.20 | <0.001 |
| FIB_max | 1.00 | 1.00, 1.00 | 0.049 |
| FIB_min | 1.00 | 1.00, 1.00 | 0.224 |
| DDimer_max | 1.00 | 1.00, 1.00 | 0.179 |
| DDimer_min | 1.00 | 1.00, 1.00 | 0.049 |
| INR_max | 1.30 | 1.02, 1.99 | 0.134 |
| INR_min | 0.73 | 0.38, 1.39 | 0.345 |
| PLT_max | 1.00 | 1.00, 1.00 | <0.001 |
| PLT_min | 1.00 | 1.00, 1.00 | 0.003 |
| HB_max | 1.11 | 1.04, 1.19 | 0.001 |
| HB_min | 0.93 | 0.86, 1.00 | 0.057 |
| WBC_max | 1.00 | 0.99, 1.01 | 0.918 |
| WBC_min | 1.04 | 1.01, 1.06 | 0.005 |
| BUN_max | 1.00 | 1.00, 1.01 | 0.111 |
| BUN_min | 1.01 | 1.00, 1.02 | 0.023 |
| Creatinine_max | 0.94 | 0.87, 1.01 | 0.087 |
| BNP_max | 1.00 | 1.00, 1.00 | 0.653 |
| Lactate_max | 1.09 | 1.04, 1.13 | <0.001 |
| Lactate_min | 1.54 | 1.33, 1.79 | <0.001 |
| HCO3_max | 0.89 | 0.86, 0.91 | <0.001 |
| HCO3_min | 1.01 | 0.97, 1.04 | 0.677 |
| PCO2_max | 1.05 | 1.04, 1.06 | <0.001 |
| PCO2_min | 0.98 | 0.97, 1.00 | 0.015 |
| PO2_max | 1.00 | 1.00, 1.00 | <0.001 |
| PO2_min | 1.00 | 0.99, 1.00 | <0.001 |
| PH_max | 17.4 | 2.80, 109 | 0.002 |
| PH_min | 3.06 | 0.68, 13.8 | 0.145 |
| Temperature_max | 0.80 | 0.72, 0.90 | <0.001 |
| Temperature_min | 0.84 | 0.76, 0.93 | <0.001 |
| Heart_Rate_max | 1.00 | 1.00, 1.00 | 0.959 |
| MAP_min | 1.0 | 0.99, 1.00 | 0.134 |
| Vasopressor |  |  |  |
| YES | 1.00 | Reference |  |
| NO | 0.82 | 0.67, 0.99 | 0.043 |
| Continuous_Renal_Replacement_Therapy |  |  |  |
| YES | 1.00 | Reference |  |
| NO | 1.10 | 0.71, 1.73 | 0.674 |
| Mechanical_ventilation |  |  |  |
| YES | 1.00 | Reference |  |
| NO | 0.46 | 0.37, 0.58 | <0.001 |
| Age | 1.02 | 1.02, 1.03 | <0.001 |
| ^1^OR = Odds Ratio, CI = Confidence Interval | | | |

Supplementary Table 13. Multivariate logistic model adjusted with unbalanced covariates (Variables other than the group listed in the table) using IPTW for 28-day mortality of cohort

| **Characteristic** | **OR**^1^ | **95% CI**^1^ | **p-value** |
| --- | --- | --- | --- |
| Group |  |  |  |
| non-SIMI | 1.00 | Reference |  |
| SIMI | 1.30 | 1.10, 1.53 | 0.002 |
| Mechanical_ventilation |  |  |  |
| YES | 1.00 | Reference |  |
| NO | 0.44 | 0.33, 0.57 | <0.001 |
| Continuous_Renal_Replacement_Therapy |  |  |  |
| YES | 1.00 | Reference |  |
| NO | 1.06 | 0.68, 1.64 | 0.809 |
| Sedative_Use |  |  |  |
| YES | 1.00 | Reference |  |
| NO | 1.49 | 1.15, 1.94 | 0.003 |
| Vasopressor |  |  |  |
| YES | 1.00 | Reference |  |
| NO | 0.72 | 0.59, 0.89 | 0.002 |
| Septic_Shock |  |  |  |
| YES | 1.00 | Reference |  |
| NO | 1.09 | 0.88, 1.34 | 0.449 |
| MAP_min | 0.99 | 0.99, 1.00 | 0.009 |
| MAP_max | 1.00 | 1.00, 1.00 | 0.159 |
| Temperature_min | 0.90 | 0.82, 0.99 | 0.031 |
| Temperature_max | 0.78 | 0.70, 0.86 | <0.001 |
| PH_min | 0.04 | 0.01, 0.13 | <0.001 |
| PO2_min | 0.99 | 0.99, 1.00 | <0.001 |
| PO2_max | 1.00 | 1.00, 1.00 | <0.001 |
| PCO2_min | 0.99 | 0.98, 1.00 | 0.062 |
| HCO3_min | 1.06 | 1.02, 1.09 | <0.001 |
| HCO3_max | 0.94 | 0.92, 0.96 | <0.001 |
| Lactate_min | 1.53 | 1.33, 1.77 | <0.001 |
| Lactate_max | 1.08 | 1.04, 1.12 | <0.001 |
| BNP_max | 1.00 | 1.00, 1.00 | 0.265 |
| Creatinine_max | 0.89 | 0.83, 0.95 | <0.001 |
| BUN_min | 1.02 | 1.01, 1.02 | <0.001 |
| BUN_max | 1.01 | 1.00, 1.01 | 0.004 |
| WBC_min | 1.05 | 1.02, 1.07 | <0.001 |
| WBC_max | 1.00 | 0.99, 1.01 | 0.824 |
| HB_min | 0.95 | 0.88, 1.02 | 0.141 |
| HB_max | 1.08 | 1.02, 1.15 | 0.011 |
| PLT_min | 1.00 | 1.00, 1.00 | 0.003 |
| PLT_max | 1.00 | 1.00, 1.00 | <0.001 |
| INR_min | 0.60 | 0.30, 1.19 | 0.149 |
| INR_max | 1.84 | 1.20, 3.10 | 0.021 |
| DDimer_min | 1.00 | 1.00, 1.00 | <0.001 |
| DDimer_max | 1.00 | 1.00, 1.00 | 0.226 |
| FIB_min | 1.00 | 1.00, 1.00 | 0.237 |
| FIB_max | 1.00 | 1.00, 1.00 | 0.51 |
| PT_min | 1.15 | 1.07, 1.23 | <0.001 |
| PT_max | 0.95 | 0.90, 0.99 | 0.061 |
| ALT_max | 1.00 | 1.00, 1.00 | 0.273 |
| AST_max | 1.00 | 1.00, 1.00 | 0.291 |
| ABL_min | 0.79 | 0.67, 0.94 | 0.007 |
| Ca_min | 1.03 | 0.91, 1.17 | 0.62 |
| Ca_max | 1.00 | 0.92, 1.09 | 0.979 |
| Na_max | 1.03 | 1.02, 1.04 | <0.001 |
| K_max | 1.15 | 1.04, 1.27 | 0.005 |
| ^1^OR = Odds Ratio, CI = Confidence Interval | | | |

Supplementary Table 14. Survey-weighted generalized linear model adjusted with Boruta selected covariates (Variables other than the group listed in the table) using IPTW for 28-day mortality of cohort

| **Characteristic** | **OR**^1^ | **95% CI**^1^ | **p-value** |
| --- | --- | --- | --- |
| Group |  |  |  |
| non-SIMI | 1.00 | Reference |  |
| SIMI | 1.35 | 1.02, 1.79 | 0.035 |
| K_max | 1.08 | 0.95, 1.22 | 0.234 |
| K_min | 1.40 | 1.02, 1.92 | 0.035 |
| Na_max | 1.04 | 1.01, 1.07 | 0.014 |
| Na_min | 0.99 | 0.97, 1.01 | 0.233 |
| Ca_max | 1.01 | 0.91, 1.13 | 0.813 |
| ABL_max | 0.76 | 0.56, 1.03 | 0.074 |
| ABL_min | 0.94 | 0.68, 1.29 | 0.698 |
| AST_max | 1.00 | 1.00, 1.00 | 0.476 |
| ALT_max | 1.00 | 1.00, 1.00 | 0.499 |
| PT_max | 0.98 | 0.95, 1.02 | 0.362 |
| PT_min | 1.13 | 1.03, 1.23 | 0.007 |
| FIB_max | 1.00 | 1.00, 1.00 | 0.184 |
| FIB_min | 1.00 | 1.00, 1.00 | 0.464 |
| DDimer_max | 1.00 | 1.00, 1.00 | 0.374 |
| DDimer_min | 1.00 | 1.00, 1.00 | 0.193 |
| INR_max | 1.30 | 0.92, 1.82 | 0.136 |
| INR_min | 0.73 | 0.25, 2.17 | 0.572 |
| PLT_max | 1.00 | 1.0, 1.00 | <0.001 |
| PLT_min | 1.00 | 1.00, 1.01 | 0.055 |
| HB_max | 1.11 | 1.01, 1.22 | 0.025 |
| HB_min | 0.93 | 0.83, 1.04 | 0.193 |
| WBC_max | 1.00 | 0.98, 1.02 | 0.94 |
| WBC_min | 1.04 | 1.00, 1.08 | 0.07 |
| BUN_max | 1.00 | 1.00, 1.01 | 0.266 |
| BUN_min | 1.01 | 1.00, 1.02 | 0.15 |
| Creatinine_max | 0.94 | 0.86, 1.03 | 0.188 |
| BNP_max | 1.00 | 1.00, 1.00 | 0.769 |
| Lactate_max | 1.09 | 1.02, 1.16 | 0.012 |
| Lactate_min | 1.54 | 1.25, 1.89 | <0.001 |
| HCO3_max | 0.89 | 0.85, 0.93 | <0.001 |
| HCO3_min | 1.01 | 0.95, 1.06 | 0.795 |
| PCO2_max | 1.05 | 1.03, 1.06 | <0.001 |
| PCO2_min | 0.98 | 0.96, 1.01 | 0.128 |
| PO2_max | 1.00 | 1.00, 1.00 | 0.005 |
| PO2_min | 1.00 | 0.99, 1.00 | 0.01 |
| PH_max | 17.4 | 1.05, 289 | 0.046 |
| PH_min | 3.06 | 0.35, 26.3 | 0.309 |
| Temperature_max | 0.80 | 0.67, 0.96 | 0.015 |
| Temperature_min | 0.84 | 0.72, 0.98 | 0.029 |
| Heart_Rate_max | 1.00 | 0.99, 1.01 | 0.975 |
| MAP_min | 1.0 | 0.99, 1.00 | 0.3 |
| Vasopressor |  |  |  |
| YES | 1.00 | Reference |  |
| NO | 0.82 | 0.60, 1.12 | 0.205 |
| Continuous_Renal_Replacement_Therapy |  |  |  |
| YES | 1.00 | Reference |  |
| NO | 1.10 | 0.58, 2.10 | 0.771 |
| Mechanical_ventilation |  |  |  |
| YES | 1.00 | Reference |  |
| NO | 0.46 | 0.33, 0.66 | <0.001 |
| Age | 1.02 | 1.01, 1.03 | <0.001 |
| ^1^OR = Odds Ratio, CI = Confidence Interval | | | |

Supplementary Table 15. Multivariate Cox model adjusted with Boruta selected covariates (Variables other than the group listed in the table) for 180-day mortality of original cohort

| **Characteristic** | **HR^1^** | **95% CI^1^** | **p-value** |
| --- | --- | --- | --- |
| Group |  |  |  |
| non-SIMI | 1.00 | Reference |  |
| SIMI | 1.29 | 1.10, 1.52 | 0.002 |
| K_max (time dependent) | 1.02 | 1.00, 1.04 | 0.034 |
| K_min (time dependent) | 0.98 | 0.94, 1.03 | 0.472 |
| Na_max (time dependent) | 1.00 | 1.00, 1.01 | 0.003 |
| Na_min (time dependent) | 1.00 | 1.00, 1.00 | 0.765 |
| Ca_max (time dependent) | 1.00 | 0.98, 1.02 | 0.761 |
| ABL_max | 0.96 | 0.83, 1.12 | 0.617 |
| ABL_min (time dependent) | 0.97 | 0.93, 1.02 | 0.198 |
| AST_max | 1.00 | 1.00, 1.00 | <0.001 |
| ALT_max | 1.00 | 1.00, 1.00 | 0.002 |
| PT_max (time dependent) | 1.00 | 1.00, 1.00 | 0.855 |
| PT_min (time dependent) | 1.00 | 0.99, 1.01 | 0.752 |
| FIB_max | 1.00 | 1.00, 1.00 | 0.168 |
| FIB_min | 1.00 | 1.00, 1.00 | 0.362 |
| DDimer_max (time dependent) | 1.00 | 1.00, 1.00 | 0.324 |
| DDimer_min | 1.00 | 1.00, 1.00 | 0.026 |
| INR_max (time dependent) | 1.01 | 0.98, 1.04 | 0.59 |
| INR_min (time dependent) | 1.07 | 0.94, 1.21 | 0.326 |
| PLT_max (time dependent) | 1.00 | 1.00, 1.00 | <0.001 |
| PLT_min (time dependent) | 1.00 | 1.00, 1.00 | 0.06 |
| HB_max (time dependent) | 1.02 | 1.01, 1.04 | 0.004 |
| HB_min (time dependent) | 0.98 | 0.97, 1.00 | 0.044 |
| WBC_max (time dependent) | 1.00 | 1.00, 1.00 | 0.445 |
| WBC_min (time dependent) | 1.01 | 1.00, 1.01 | 0.011 |
| BUN_max (time dependent) | 1.00 | 1.00, 1.00 | 0.11 |
| BUN_min (time dependent) | 1.00 | 1.00, 1.00 | 0.001 |
| Creatinine_max | 0.96 | 0.91, 1.01 | 0.091 |
| BNP_max (time dependent) | 1.00 | 1.00, 1.00 | 0.016 |
| Lactate_max | 1.05 | 1.03, 1.08 | <0.001 |
| Lactate_min | 1.18 | 1.12, 1.23 | <0.001 |
| HCO3_max (time dependent) | 0.98 | 0.98, 0.99 | <0.001 |
| HCO3_min | 1.01 | 0.98, 1.03 | 0.669 |
| PCO2_max | 1.02 | 1.02, 1.03 | <0.001 |
| PCO2_min | 0.99 | 0.98, 1.01 | 0.392 |
| PO2_max | 1.00 | 1.00, 1.00 | 0.085 |
| PO2_min (time dependent) | 1.00 | 1.00, 1.00 | 0.008 |
| PH_max (time dependent) | 1.12 | 0.76, 1.66 | 0.567 |
| PH_min (time dependent) | 0.99 | 0.73, 1.35 | 0.964 |
| Temperature_max | 0.87 | 0.80, 0.94 | <0.001 |
| Temperature_min | 0.89 | 0.83, 0.96 | 0.003 |
| Heart_Rate_max (time dependent) | 1.00 | 1.00, 1.00 | 0.123 |
| MAP_min (time dependent) | 1.00 | 1.00, 1.00 | 0.009 |
| Vasopressor (time dependent) |  |  |  |
| YES | 1.00 | Reference |  |
| NO | 0.97 | 0.93, 1.02 | 0.218 |
| Continuous_Renal_Replacement_Therapy |  |  |  |
| YES | 1.00 | Reference |  |
| NO | 0.87 | 0.65, 1.17 | 0.367 |
| Mechanical_ventilation (time dependent) |  |  |  |
| YES | 1.00 | Reference |  |
| NO | 0.89 | 0.84, 0.93 | <0.001 |
| Age (time dependent) | 1.00 | 1.00, 1.01 | <0.001 |
| ^1^HR = Hazard Ratio, CI = Confidence Interval | | | |

Supplementary Table 16. Multivariate Cox model adjusted with unbalanced covariates (Variables other than the group listed in the table) for 180-day mortality of original cohort

| **Characteristic** | **HR^1^** | **95% CI^1^** | **p-value** |
| --- | --- | --- | --- |
| Group |  |  |  |
| non-SIMI | 1.00 | Reference |  |
| SIMI | 1.28 | 1.09, 1.50 | 0.003 |
| Mechanical_ventilation (time dependent) |  |  |  |
| YES | 1.00 | Reference |  |
| NO | 0.84 | 0.79, 0.90 | <0.001 |
| Continuous_Renal_Replacement_Therapy |  |  |  |
| YES | 1.00 | Reference |  |
| NO | 0.85 | 0.63, 1.14 | 0.269 |
| Sedative_Use (time dependent) |  |  |  |
| YES | 1.00 | Reference |  |
| NO | 1.14 | 1.07, 1.21 | <0.001 |
| Vasopressor (time dependent) |  |  |  |
| YES | 1.00 | Reference |  |
| NO | 0.93 | 0.89, 0.98 | 0.008 |
| Septic_Shock (time dependent) |  |  |  |
| YES | 1.00 | Reference |  |
| NO | 0.98 | 0.94, 1.03 | 0.461 |
| MAP_min (time dependent) | 1.00 | 1.00, 1.00 | <0.001 |
| MAP_max (time dependent) | 1.00 | 1.00, 1.00 | 0.6 |
| Temperature_min | 0.90 | 0.84, 0.96 | 0.003 |
| Temperature_max | 0.87 | 0.80, 0.94 | <0.001 |
| PH_min (time dependent) | 0.52 | 0.41, 0.65 | <0.001 |
| PO2_min (time dependent) | 1.00 | 1.00, 1.00 | <0.001 |
| PO2_max | 1.00 | 1.00, 1.00 | 0.003 |
| PCO2_min (time dependent) | 1.00 | 1.00, 1.00 | 0.957 |
| HCO3_min | 1.04 | 1.01, 1.06 | 0.009 |
| HCO3_max (time dependent) | 0.99 | 0.98, 0.99 | <0.001 |
| Lactate_min | 1.18 | 1.13, 1.23 | <0.001 |
| Lactate_max | 1.05 | 1.02, 1.08 | <0.001 |
| BNP_max (time dependent) | 1.00 | 1.00, 1.00 | 0.016 |
| Creatinine_max | 0.93 | 0.89, 0.98 | 0.005 |
| BUN_min (time dependent) | 1.00 | 1.00, 1.00 | <0.001 |
| BUN_max (time dependent) | 1.00 | 1.00, 1.00 | 0.024 |
| WBC_min (time dependent) | 1.01 | 1.00, 1.01 | <0.001 |
| WBC_max (time dependent) | 1.00 | 1.00, 1.00 | 0.128 |
| HB_min (time dependent) | 0.98 | 0.96, 1.00 | 0.023 |
| HB_max (time dependent) | 1.02 | 1.01, 1.04 | 0.001 |
| PLT_min (time dependent) | 1.00 | 1.00, 1.00 | 0.024 |
| PLT_max (time dependent) | 1.00 | 1.00, 1.00 | <0.001 |
| INR_min (time dependent) | 1.06 | 0.93, 1.20 | 0.392 |
| INR_max (time dependent) | 1.02 | 0.99, 1.06 | 0.166 |
| DDimer_min | 1.00 | 1.00, 1.00 | 0.301 |
| DDimer_max (time dependent) | 1.00 | 1.00, 1.00 | 0.273 |
| FIB_min | 1.00 | 1.00, 1.00 | 0.388 |
| FIB_max | 1.00 | 1.00, 1.00 | 0.448 |
| PT_min (time dependent) | 1.00 | 0.99, 1.01 | 0.98 |
| PT_max (time dependent) | 1.00 | 0.99, 1.00 | 0.419 |
| ALT_max | 1.00 | 1.00, 1.00 | <0.001 |
| AST_max | 1.00 | 1.00, 1.00 | <0.001 |
| ABL_min (time dependent) | 0.96 | 0.92, 1.00 | 0.042 |
| Ca_min | 1.02 | 0.93, 1.12 | 0.718 |
| Ca_max (time dependent) | 1.00 | 0.98, 1.02 | 0.646 |
| Na_max (time dependent) | 1.01 | 1.00, 1.01 | <0.001 |
| K_max (time dependent) | 1.02 | 1.00, 1.04 | 0.022 |
| ^1^HR = Hazard Ratio, CI = Confidence Interval | | | |

Supplementary Table 17. Multivariate Cox model adjusted with Boruta selected covariates (Variables other than the group listed in the table) using IPTW for 180-day mortality of cohort

| **Characteristic** | **HR^1^** | **95% CI^1^** | **p-value** |
| --- | --- | --- | --- |
| Group |  |  |  |
| non-SIMI | 1.00 | Reference |  |
| SIMI | 1.20 | 1.01, 1.44 | 0.042 |
| K_max (time dependent) | 1.02 | 1.00, 1.04 | 0.05 |
| K_min (time dependent) | 1.01 | 0.95, 1.07 | 0.802 |
| Na_max | 1.01 | 1.00, 1.02 | 0.025 |
| Na_min (time dependent) | 1.00 | 1.00, 1.00 | 0.772 |
| Ca_max (time dependent) | 1.00 | 0.97, 1.02 | 0.754 |
| ABL_max (time dependent) | 0.99 | 0.93, 1.05 | 0.689 |
| ABL_min (time dependent) | 0.98 | 0.92, 1.03 | 0.387 |
| AST_max | 1.00 | 1.00, 1.00 | <0.001 |
| ALT_max | 1.00 | 1.00, 1.00 | 0.002 |
| PT_max (time dependent) | 1.00 | 1.00, 1.00 | 0.808 |
| PT_min (time dependent) | 1.00 | 0.99, 1.01 | 0.722 |
| FIB_max | 1.00 | 1.00, 1.00 | 0.344 |
| FIB_min (time dependent) | 1.00 | 1.00, 1.00 | 0.697 |
| DDimer_max (time dependent) | 1.00 | 1.00, 1.00 | 0.547 |
| DDimer_min | 1.00 | 1.00, 1.00 | 0.335 |
| INR_max (time dependent) | 1.02 | 0.98, 1.05 | 0.331 |
| INR_min (time dependent) | 1.08 | 0.95, 1.23 | 0.252 |
| PLT_max (time dependent) | 1.00 | 1.00, 1.00 | <0.001 |
| PLT_min (time dependent) | 1.00 | 1.00, 1.00 | 0.275 |
| HB_max (time dependent) | 1.02 | 1.01, 1.04 | 0.004 |
| HB_min (time dependent) | 0.98 | 0.96, 1.00 | 0.018 |
| WBC_max (time dependent) | 1.00 | 1.00, 1.00 | 0.332 |
| WBC_min (time dependent) | 1.01 | 1.00, 1.01 | 0.011 |
| BUN_max (time dependent) | 1.00 | 1.00, 1.00 | 0.063 |
| BUN_min (time dependent) | 1.00 | 1.00, 1.00 | 0.006 |
| Creatinine_max (time dependent) | 0.98 | 0.97, 1.00 | 0.071 |
| BNP_max (time dependent) | 1.00 | 1.00, 1.00 | 0.011 |
| Lactate_max | 1.05 | 1.02, 1.09 | <0.001 |
| Lactate_min | 1.18 | 1.12, 1.25 | <0.001 |
| HCO3_max (time dependent) | 0.98 | 0.97, 0.99 | <0.001 |
| HCO3_min (time dependent) | 1.00 | 0.99, 1.01 | 0.467 |
| PCO2_max | 1.03 | 1.02, 1.03 | <0.001 |
| PCO2_min | 0.99 | 0.98, 1.01 | 0.374 |
| PO2_max | 1.00 | 1.00, 1.00 | 0.014 |
| PO2_min (time dependent) | 1.00 | 1.00, 1.00 | 0.008 |
| PH_max (time dependent) | 1.26 | 0.76, 2.09 | 0.361 |
| PH_min (time dependent) | 1.04 | 0.71, 1.52 | 0.836 |
| Temperature_max | 0.83 | 0.75, 0.92 | <0.001 |
| Temperature_min | 0.91 | 0.83, 0.99 | 0.027 |
| Heart_Rate_max (time dependent) | 1.00 | 1.00, 1.00 | 0.346 |
| MAP_min (time dependent) | 1.00 | 1.00, 1.00 | 0.004 |
| Vasopressor (time dependent) |  |  |  |
| YES | 1.00 | Reference |  |
| NO | 0.96 | 0.91, 1.02 | 0.201 |
| Continuous_Renal_Replacement_Therapy |  |  |  |
| YES | 1.00 | Reference |  |
| NO | 0.87 | 0.61, 1.22 | 0.409 |
| Mechanical_ventilation (time dependent) |  |  |  |
| YES | 1.00 | Reference |  |
| NO | 0.91 | 0.86, 0.97 | 0.005 |
| Age (time dependent) | 1.00 | 1.00, 1.01 | <0.001 |
| ^1^HR = Hazard Ratio, CI = Confidence Interval | | | |

Supplementary Table 18. Multivariate Cox model adjusted with unbalanced covariates (Variables other than the group listed in the table) using IPTW for 180-day mortality of cohort

| **Characteristic** | **OR**^1^ | **95% CI**^1^ | **p-value** |
| --- | --- | --- | --- |
| Group |  |  |  |
| non-SIMI | 1.00 | Reference |  |
| SIMI | 1.17 | 1.00, 1.35 | 0.044 |
| Mechanical_ventilation (time dependent) |  |  |  |
| YES | 1.00 | Reference |  |
| NO | 0.58 | 0.46, 0.74 | <0.001 |
| Continuous_Renal_Replacement_Therapy |  |  |  |
| YES | 1.00 | Reference |  |
| NO | 0.88 | 0.58, 1.32 | 0.532 |
| Sedative_Use (time dependent) |  |  |  |
| YES | 1.00 | Reference |  |
| NO | 1.83 | 1.44, 2.32 | <0.001 |
| Vasopressor (time dependent) |  |  |  |
| YES | 1.00 | Reference |  |
| NO | 0.72 | 0.60, 0.86 | <0.001 |
| Septic_Shock (time dependent) |  |  |  |
| YES | 1.00 | Reference |  |
| NO | 0.99 | 0.82, 1.19 | 0.884 |
| MAP_min (time dependent) | 0.99 | 0.98, 1.00 | <0.001 |
| MAP_max (time dependent) | 1.00 | 1.00, 1.00 | 0.487 |
| Temperature_min | 0.90 | 0.83, 0.99 | 0.028 |
| Temperature_max (time dependent) | 0.75 | 0.68, 0.82 | <0.001 |
| PH_min (time dependent) | 0.17 | 0.06, 0.45 | <0.001 |
| PO2_min (time dependent) | 0.99 | 0.99, 1.00 | <0.001 |
| PO2_max | 1.00 | 1.00, 1.00 | <0.001 |
| PCO2_min | 0.99 | 0.98, 1.00 | 0.248 |
| HCO3_min | 1.02 | 0.99, 1.05 | 0.29 |
| HCO3_max (time dependent) | 0.98 | 0.96, 1.00 | 0.136 |
| Lactate_min | 1.49 | 1.31, 1.70 | <0.001 |
| Lactate_max | 1.07 | 1.03, 1.11 | <0.001 |
| BNP_max (time dependent) | 1.00 | 1.00, 1.00 | 0.188 |
| Creatinine_max (time dependent) | 0.89 | 0.83, 0.94 | <0.001 |
| BUN_min (time dependent) | 1.01 | 1.01, 1.02 | <0.001 |
| BUN_max (time dependent) | 1.01 | 1.01, 1.01 | <0.001 |
| WBC_min (time dependent) | 1.04 | 1.01, 1.06 | 0.001 |
| WBC_max (time dependent) | 0.99 | 0.98, 1.00 | 0.07 |
| HB_min (time dependent) | 0.93 | 0.87, 1.00 | 0.038 |
| HB_max (time dependent) | 1.05 | 0.99, 1.11 | 0.113 |
| PLT_min (time dependent) | 1.00 | 1.00, 1.00 | 0.083 |
| PLT_max | 1.00 | 1.00, 1.00 | <0.001 |
| INR_min (time dependent) | 1.00 | 0.56, 1.80 | 0.989 |
| INR_max (time dependent) | 1.39 | 1.13, 1.95 | 0.014 |
| DDimer_min | 1.00 | 1.00, 1.00 | <0.001 |
| DDimer_max (time dependent) | 1.00 | 1.00, 1.00 | 0.58 |
| FIB_min (time dependent) | 1.00 | 1.00, 1.00 | 0.97 |
| FIB_max | 1.00 | 1.00, 1.00 | 0.709 |
| PT_min (time dependent) | 1.10 | 1.04, 1.17 | <0.001 |
| PT_max (time dependent) | 0.98 | 0.94, 1.00 | 0.069 |
| ALT_max | 1.00 | 1.00, 1.00 | 0.042 |
| AST_max | 1.00 | 1.00, 1.00 | 0.282 |
| ABL_min (time dependent) | 0.79 | 0.68, 0.92 | 0.002 |
| Ca_min | 1.09 | 0.97, 1.22 | 0.137 |
| Ca_max (time dependent) | 1.00 | 0.92, 1.08 | 0.961 |
| Na_max | 1.03 | 1.02, 1.04 | <0.001 |
| K_max (time dependent) | 1.10 | 1.01, 1.21 | 0.03 |
| ^1^OR = Odds Ratio, CI = Confidence Interval | | | |

Supplementary Table 19. Survey-weighted Cox model adjusted with Boruta selected covariates (Variables other than the group listed in the table) using IPTW for 180-day mortality of cohort

| **Characteristic** | **HR^1^** | **95% CI^1^** | **p-value** |
| --- | --- | --- | --- |
| Group |  |  |  |
| non-SIMI | 1.00 | Reference |  |
| SIMI | 1.21 | 1.01, 1.44 | 0.036 |
| K_max (time dependent) | 1.02 | 1.00, 1.04 | 0.085 |
| K_min (time dependent) | 1.01 | 0.96, 1.07 | 0.613 |
| Na_max (time dependent) | 1.00 | 1.00, 1.01 | 0.02 |
| Na_min (time dependent) | 1.00 | 1.00, 1.00 | 0.833 |
| Ca_max (time dependent) | 1.00 | 0.98, 1.02 | 0.849 |
| ABL_max | 0.90 | 0.75, 1.09 | 0.283 |
| ABL_min (time dependent) | 0.99 | 0.93, 1.04 | 0.608 |
| AST_max | 1.00 | 1.00, 1.00 | <0.001 |
| ALT_max | 1.00 | 1.00, 1.00 | 0.003 |
| PT_max (time dependent) | 1.00 | 1.00, 1.00 | 0.797 |
| PT_min (time dependent) | 1.00 | 0.99, 1.01 | 0.763 |
| FIB_max | 1.00 | 1.00, 1.00 | 0.275 |
| FIB_min | 1.00 | 1.00, 1.00 | 0.565 |
| DDimer_max (time dependent) | 1.00 | 1.00, 1.00 | 0.568 |
| DDimer_min | 1.00 | 1.00, 1.00 | 0.357 |
| INR_max (time dependent) | 1.02 | 0.99, 1.05 | 0.302 |
| INR_min (time dependent) | 1.08 | 0.95, 1.22 | 0.265 |
| PLT_max (time dependent) | 1.00 | 1.00, 1.00 | <0.001 |
| PLT_min (time dependent) | 1.00 | 1.00, 1.00 | 0.28 |
| HB_max (time dependent) | 1.02 | 1.01, 1.04 | 0.004 |
| HB_min (time dependent) | 0.98 | 0.96, 1.00 | 0.021 |
| WBC_max (time dependent) | 1.00 | 1.00, 1.00 | 0.314 |
| WBC_min (time dependent) | 1.01 | 1.00, 1.01 | 0.012 |
| BUN_max (time dependent) | 1.00 | 1.00, 1.00 | 0.073 |
| BUN_min (time dependent) | 1.00 | 1.00, 1.00 | 0.011 |
| Creatinine_max | 0.95 | 0.89, 1.01 | 0.077 |
| BNP_max (time dependent) | 1.00 | 1.00, 1.00 | 0.014 |
| Lactate_max | 1.05 | 1.02, 1.08 | 0.001 |
| Lactate_min (time dependent) | 1.06 | 1.04, 1.07 | <0.001 |
| HCO3_max (time dependent) | 0.98 | 0.97, 0.99 | <0.001 |
| HCO3_min | 1.00 | 0.97, 1.03 | 0.846 |
| PCO2_max | 1.03 | 1.02, 1.03 | <0.001 |
| PCO2_min | 1.00 | 0.98, 1.01 | 0.489 |
| PO2_max | 1.00 | 1.00, 1.00 | 0.009 |
| PO2_min (time dependent) | 1.00 | 1.00, 1.00 | 0.007 |
| PH_max (time dependent) | 1.27 | 0.77, 2.10 | 0.341 |
| PH_min (time dependent) | 1.11 | 0.76, 1.61 | 0.592 |
| Temperature_max | 0.84 | 0.76, 0.92 | <0.001 |
| Temperature_min | 0.91 | 0.83, 0.99 | 0.025 |
| Heart_Rate_max (time dependent) | 1.00 | 1.00, 1.00 | 0.366 |
| MAP_min (time dependent) | 1.00 | 1.00, 1.00 | 0.005 |
| Vasopressor (time dependent) |  |  |  |
| YES | 1.00 | Reference |  |
| NO | 0.97 | 0.92, 1.02 | 0.247 |
| Continuous_Renal_Replacement_Therapy |  |  |  |
| YES | 1.00 | Reference |  |
| NO | 0.87 | 0.62, 1.23 | 0.444 |
| Mechanical_ventilation (time dependent) |  |  |  |
| YES | 1.00 | Reference |  |
| NO | 0.91 | 0.86, 0.97 | 0.004 |
| Age (time dependent) | 1.00 | 1.00, 1.01 | <0.001 |
| ^1^HR = Hazard Ratio, CI = Confidence Interval | | | |

Supplementary Table 20. Multivariate Cox model adjusted with Boruta selected covariates (Variables other than the group listed in the table) for 1-year mortality of original cohort

| **Characteristic** | **HR^1^** | **95% CI^1^** | **p-value** |
| --- | --- | --- | --- |
| Group (time dependent) |  |  |  |
| non-SIMI | 1.00 | Reference |  |
| SIMI | 1.04 | 1.00, 1.09 | 0.047 |
| K_max (time dependent) | 1.02 | 1.00, 1.04 | 0.058 |
| K_min (time dependent) | 0.98 | 0.94, 1.02 | 0.351 |
| Na_max | 1.01 | 1.00, 1.02 | 0.028 |
| Na_min (time dependent) | 1.00 | 1.00, 1.00 | 0.714 |
| Ca_max (time dependent) | 1.00 | 0.98, 1.01 | 0.761 |
| ABL_max | 1.00 | 0.86, 1.16 | 0.991 |
| ABL_min (time dependent) | 0.96 | 0.92, 1.00 | 0.07 |
| AST_max | 1.00 | 1.00, 1.00 | <0.001 |
| ALT_max | 1.00 | 1.00, 1.00 | 0.002 |
| PT_max (time dependent) | 1.00 | 1.00, 1.00 | 0.929 |
| PT_min (time dependent) | 1.00 | 0.99, 1.01 | 0.867 |
| FIB_max | 1.00 | 1.00, 1.00 | 0.06 |
| FIB_min (time dependent) | 1.00 | 1.00, 1.00 | 0.703 |
| DDimer_max (time dependent) | 1.00 | 1.00, 1.00 | 0.515 |
| DDimer_min (time dependent) | 1.00 | 1.00, 1.00 | 0.25 |
| INR_max (time dependent) | 1.01 | 0.98, 1.04 | 0.59 |
| INR_min (time dependent) | 1.04 | 0.92, 1.16 | 0.534 |
| PLT_max (time dependent) | 1.00 | 1.00, 1.00 | <0.001 |
| PLT_min (time dependent) | 1.00 | 1.00, 1.00 | 0.134 |
| HB_max (time dependent) | 1.01 | 1.00, 1.02 | 0.152 |
| HB_min (time dependent) | 0.99 | 0.98, 1.01 | 0.25 |
| WBC_max (time dependent) | 1.00 | 1.00, 1.00 | 0.364 |
| WBC_min (time dependent) | 1.01 | 1.00, 1.01 | 0.014 |
| BUN_max (time dependent) | 1.00 | 1.00, 1.00 | 0.043 |
| BUN_min (time dependent) | 1.00 | 1.00, 1.00 | 0.004 |
| Creatinine_max | 0.97 | 0.92, 1.02 | 0.199 |
| BNP_max (time dependent) | 1.00 | 1.00, 1.00 | 0.188 |
| Lactate_max | 1.06 | 1.03, 1.09 | <0.001 |
| Lactate_min | 1.19 | 1.14, 1.25 | <0.001 |
| HCO3_max (time dependent) | 0.98 | 0.98, 0.99 | <0.001 |
| HCO3_min | 0.99 | 0.97, 1.02 | 0.522 |
| PCO2_max | 1.03 | 1.02, 1.03 | <0.001 |
| PCO2_min | 1.00 | 0.98, 1.01 | 0.386 |
| PO2_max | 1.00 | 1.00, 1.00 | 0.026 |
| PO2_min (time dependent) | 1.00 | 1.00, 1.00 | <0.001 |
| PH_max (time dependent) | 1.07 | 0.75, 1.53 | 0.714 |
| PH_min (time dependent) | 1.21 | 0.92, 1.59 | 0.183 |
| Temperature_max | 0.87 | 0.81, 0.95 | <0.001 |
| Temperature_min | 0.89 | 0.83, 0.96 | 0.001 |
| Heart_Rate_max (time dependent) | 1.00 | 1.00, 1.00 | 0.096 |
| MAP_min (time dependent) | 1.00 | 1.00, 1.00 | 0.015 |
| Vasopressor (time dependent) |  |  |  |
| YES | 1.00 | Reference |  |
| NO | 0.97 | 0.93, 1.01 | 0.104 |
| Continuous_Renal_Replacement_Therapy |  |  |  |
| YES | 1.00 | Reference |  |
| NO | 0.88 | 0.66, 1.17 | 0.383 |
| Mechanical_ventilation (time dependent) |  |  |  |
| YES | 1.00 | Reference |  |
| NO | 0.91 | 0.87, 0.96 | <0.001 |
| Age (time dependent) | 1.00 | 1.00, 1.01 | <0.001 |
| ^1^HR = Hazard Ratio, CI = Confidence Interval | | | |

Supplementary Table 21. Multivariate Cox model adjusted with unbalanced covariates (Variables other than the group listed in the table) for 1-year mortality of original cohort

| **Characteristic** | **HR^1^** | **95% CI^1^** | **p-value** |
| --- | --- | --- | --- |
| Group (time dependent) |  |  |  |
| non-SIMI | 1.00 | Reference |  |
| SIMI | 1.04 | 1.00, 1.08 | 0.079 |
| Mechanical_ventilation (time dependent) |  |  |  |
| YES | 1.00 | Reference |  |
| NO | 0.88 | 0.83, 0.93 | <0.001 |
| Continuous_Renal_Replacement_Therapy |  |  |  |
| YES | 1.00 | Reference |  |
| NO | 0.86 | 0.64, 1.15 | 0.308 |
| Sedative_Use (time dependent) |  |  |  |
| YES | 1.00 | Reference |  |
| NO | 1.12 | 1.06, 1.19 | <0.001 |
| Vasopressor (time dependent) |  |  |  |
| YES | 1.00 | Reference |  |
| NO | 0.94 | 0.89, 0.98 | 0.005 |
| Septic_Shock (time dependent) |  |  |  |
| YES | 1.00 | Reference |  |
| NO | 0.97 | 0.93, 1.01 | 0.134 |
| MAP_min (time dependent) | 1.00 | 1.00, 1.00 | <0.001 |
| MAP_max (time dependent) | 1.00 | 1.00, 1.00 | 0.256 |
| Temperature_min | 0.89 | 0.83, 0.96 | 0.001 |
| Temperature_max | 0.87 | 0.81, 0.94 | <0.001 |
| PH_min (time dependent) | 0.62 | 0.50, 0.77 | <0.001 |
| PO2_min (time dependent) | 1.00 | 1.00, 1.00 | <0.001 |
| PO2_max | 1.00 | 1.00, 1.00 | <0.001 |
| PCO2_min (time dependent) | 1.00 | 1.00, 1.00 | 0.791 |
| HCO3_min | 1.02 | 1.00, 1.05 | 0.08 |
| HCO3_max (time dependent) | 0.99 | 0.99, 1.00 | <0.001 |
| Lactate_min | 1.19 | 1.14, 1.24 | <0.001 |
| Lactate_max | 1.05 | 1.03, 1.08 | <0.001 |
| BNP_max (time dependent) | 1.00 | 1.00, 1.00 | 0.217 |
| Creatinine_max | 0.94 | 0.90, 0.99 | 0.012 |
| BUN_min (time dependent) | 1.00 | 1.00, 1.00 | <0.001 |
| BUN_max (time dependent) | 1.00 | 1.00, 1.00 | 0.005 |
| WBC_min (time dependent) | 1.01 | 1.00, 1.01 | <0.001 |
| WBC_max (time dependent) | 1.00 | 1.00, 1.00 | 0.055 |
| HB_min (time dependent) | 0.99 | 0.97, 1.00 | 0.138 |
| HB_max (time dependent) | 1.01 | 1.00, 1.03 | 0.068 |
| PLT_min (time dependent) | 1.00 | 1.00, 1.00 | 0.061 |
| PLT_max (time dependent) | 1.00 | 1.00, 1.00 | <0.001 |
| INR_min (time dependent) | 1.03 | 0.92, 1.16 | 0.597 |
| INR_max (time dependent) | 1.03 | 0.99, 1.06 | 0.122 |
| DDimer_min (time dependent) | 1.00 | 1.00, 1.00 | 0.809 |
| DDimer_max (time dependent) | 1.00 | 1.00, 1.00 | 0.428 |
| FIB_min (time dependent) | 1.00 | 1.00, 1.00 | 0.753 |
| FIB_max | 1.00 | 1.00, 1.00 | 0.243 |
| PT_min (time dependent) | 1.00 | 0.99, 1.01 | 0.643 |
| PT_max (time dependent) | 1.00 | 0.99, 1.00 | 0.274 |
| ALT_max | 1.00 | 1.00, 1.00 | <0.001 |
| AST_max | 1.00 | 1.00, 1.00 | <0.001 |
| ABL_min (time dependent) | 0.96 | 0.93, 0.99 | 0.025 |
| Ca_min | 1.03 | 0.94, 1.12 | 0.592 |
| Ca_max (time dependent) | 1.00 | 0.98, 1.01 | 0.63 |
| Na_max | 1.02 | 1.01, 1.03 | <0.001 |
| K_max (time dependent) | 1.02 | 1.00, 1.03 | 0.052 |
| ^1^HR = Hazard Ratio, CI = Confidence Interval | | | |

Supplementary Table 22. Multivariate Cox model adjusted with Boruta selected covariates (Variables other than the group listed in the table) using IPTW for 1-year mortality of cohort

| **Characteristic** | **HR^1^** | **95% CI^1^** | **p-value** |
| --- | --- | --- | --- |
| Group |  |  |  |
| non-SIMI | 1.00 | Reference |  |
| SIMI | 1.15 | 0.97, 1.37 | 0.107 |
| K_max (time dependent) | 1.02 | 1.00, 1.04 | 0.104 |
| K_min (time dependent) | 0.99 | 0.94, 1.04 | 0.662 |
| Na_max | 1.01 | 1.00, 1.02 | 0.155 |
| Na_min (time dependent) | 1.00 | 1.00, 1.00 | 0.758 |
| Ca_max (time dependent) | 1.00 | 0.98, 1.02 | 0.758 |
| ABL_max (time dependent) | 1.01 | 0.96, 1.07 | 0.639 |
| ABL_min (time dependent) | 0.96 | 0.91, 1.01 | 0.097 |
| AST_max | 1.00 | 1.00, 1.00 | <0.001 |
| ALT_max (time dependent) | 1.00 | 1.00, 1.00 | 0.091 |
| PT_max (time dependent) | 1.00 | 1.00, 1.00 | 0.74 |
| PT_min (time dependent) | 1.00 | 0.99, 1.01 | 0.965 |
| FIB_max | 1.00 | 1.00, 1.00 | 0.207 |
| FIB_min (time dependent) | 1.00 | 1.00, 1.00 | 0.969 |
| DDimer_max (time dependent) | 1.00 | 1.00, 1.00 | 0.723 |
| DDimer_min (time dependent) | 1.00 | 1.00, 1.00 | 0.754 |
| INR_max (time dependent) | 1.02 | 0.99, 1.04 | 0.276 |
| INR_min (time dependent) | 1.05 | 0.94, 1.18 | 0.385 |
| PLT_max (time dependent) | 1.00 | 1.00, 1.00 | <0.001 |
| PLT_min (time dependent) | 1.00 | 1.00, 1.00 | 0.337 |
| HB_max (time dependent) | 1.01 | 1.00, 1.03 | 0.057 |
| HB_min (time dependent) | 0.99 | 0.97, 1.01 | 0.161 |
| WBC_max | 1.00 | 0.99, 1.01 | 0.398 |
| WBC_min (time dependent) | 1.01 | 1.00, 1.01 | 0.02 |
| BUN_max (time dependent) | 1.00 | 1.00, 1.00 | 0.055 |
| BUN_min (time dependent) | 1.00 | 1.00, 1.00 | 0.006 |
| Creatinine_max | 0.96 | 0.91, 1.02 | 0.192 |
| BNP_max (time dependent) | 1.00 | 1.00, 1.00 | 0.226 |
| Lactate_max | 1.06 | 1.03, 1.10 | <0.001 |
| Lactate_min | 1.19 | 1.13, 1.25 | <0.001 |
| HCO3_max (time dependent) | 0.98 | 0.97, 0.99 | <0.001 |
| HCO3_min (time dependent) | 1.00 | 0.99, 1.01 | 0.748 |
| PCO2_max | 1.03 | 1.02, 1.03 | <0.001 |
| PCO2_min | 0.99 | 0.98, 1.01 | 0.241 |
| PO2_max | 1.00 | 1.00, 1.00 | 0.007 |
| PO2_min (time dependent) | 1.00 | 1.00, 1.00 | <0.001 |
| PH_max (time dependent) | 1.11 | 0.69, 1.78 | 0.666 |
| PH_min (time dependent) | 1.26 | 0.90, 1.77 | 0.181 |
| Temperature_max | 0.83 | 0.75, 0.91 | <0.001 |
| Temperature_min | 0.91 | 0.83, 0.98 | 0.02 |
| Heart_Rate_max (time dependent) | 1.00 | 1.00, 1.00 | 0.18 |
| MAP_min (time dependent) | 1.00 | 1.00, 1.00 | 0.004 |
| Vasopressor (time dependent) |  |  |  |
| YES | 1.00 | Reference |  |
| NO | 0.96 | 0.91, 1.01 | 0.109 |
| Continuous_Renal_Replacement_Therapy |  |  |  |
| YES | 1.00 | Reference |  |
| NO | 0.89 | 0.63, 1.24 | 0.493 |
| Mechanical_ventilation (time dependent) |  |  |  |
| YES | 1.00 | Reference |  |
| NO | 0.94 | 0.89, 1.00 | 0.04 |
| Age (time dependent) | 1.00 | 1.00, 1.01 | <0.001 |
| ^1^HR = Hazard Ratio, CI = Confidence Interval | | | |

Supplementary Table 23. Multivariate Cox model adjusted with unbalanced covariates (Variables other than the group listed in the table) using IPTW for 1-year mortality of cohort

| **Characteristic** | **HR^1^** | **95% CI^1^** | **p-value** |
| --- | --- | --- | --- |
| Group |  |  |  |
| non-SIMI | 1.00 | Reference |  |
| SIMI | 1.12 | 0.95, 1.33 | 0.187 |
| Mechanical_ventilation (time dependent) |  |  |  |
| YES | 1.00 | Reference |  |
| NO | 0.90 | 0.84, 0.96 | 0.002 |
| Continuous_Renal_Replacement_Therapy |  |  |  |
| YES | 1.00 | Reference |  |
| NO | 0.88 | 0.63, 1.24 | 0.473 |
| Sedative_Use (time dependent) |  |  |  |
| YES | 1.00 | Reference |  |
| NO | 1.13 | 1.06, 1.22 | <0.001 |
| Vasopressor (time dependent) |  |  |  |
| YES | 1.00 | Reference |  |
| NO | 0.93 | 0.87, 0.99 | 0.014 |
| Septic_Shock (time dependent) |  |  |  |
| YES | 1.00 | Reference |  |
| NO | 0.98 | 0.93, 1.04 | 0.517 |
| MAP_min (time dependent) | 1.00 | 1.00, 1.00 | <0.001 |
| MAP_max (time dependent) | 1.00 | 1.00, 1.00 | 0.305 |
| Temperature_min | 0.91 | 0.85, 0.99 | 0.023 |
| Temperature_max (time dependent) | 0.95 | 0.93, 0.97 | <0.001 |
| PH_min (time dependent) | 0.65 | 0.50, 0.85 | 0.002 |
| PO2_min (time dependent) | 1.00 | 1.00, 1.00 | <0.001 |
| PO2_max | 1.00 | 1.00, 1.00 | <0.001 |
| PCO2_min (time dependent) | 1.00 | 1.00, 1.00 | 0.902 |
| HCO3_min (time dependent) | 1.01 | 1.00, 1.02 | 0.014 |
| HCO3_max (time dependent) | 0.99 | 0.98, 1.00 | <0.001 |
| Lactate_min | 1.19 | 1.14, 1.24 | <0.001 |
| Lactate_max | 1.06 | 1.03, 1.09 | <0.001 |
| BNP_max (time dependent) | 1.00 | 1.00, 1.00 | 0.271 |
| Creatinine_max | 0.94 | 0.89, 0.99 | 0.019 |
| BUN_min (time dependent) | 1.00 | 1.00, 1.00 | <0.001 |
| BUN_max (time dependent) | 1.00 | 1.00, 1.00 | 0.007 |
| WBC_min (time dependent) | 1.01 | 1.00, 1.01 | 0.001 |
| WBC_max | 0.99 | 0.98, 1.00 | 0.13 |
| HB_min (time dependent) | 0.99 | 0.97, 1.01 | 0.163 |
| HB_max (time dependent) | 1.02 | 1.00, 1.03 | 0.038 |
| PLT_min (time dependent) | 1.00 | 1.00, 1.00 | 0.248 |
| PLT_max (time dependent) | 1.00 | 1.00, 1.00 | <0.001 |
| INR_min (time dependent) | 1.04 | 0.93, 1.17 | 0.462 |
| INR_max (time dependent) | 1.03 | 1.00, 1.06 | 0.028 |
| DDimer_min (time dependent) | 1.00 | 1.00, 1.00 | 0.453 |
| DDimer_max (time dependent) | 1.00 | 1.00, 1.00 | 0.673 |
| FIB_min (time dependent) | 1.00 | 1.00, 1.00 | 0.77 |
| FIB_max | 1.00 | 1.00, 1.00 | 0.849 |
| PT_min (time dependent) | 1.00 | 0.99, 1.01 | 0.698 |
| PT_max (time dependent) | 1.00 | 0.99, 1.00 | 0.114 |
| ALT_max (time dependent) | 1.00 | 1.00, 1.00 | 0.047 |
| AST_max | 1.00 | 1.00, 1.00 | <0.001 |
| ABL_min (time dependent) | 0.97 | 0.93, 1.01 | 0.147 |
| Ca_min | 0.98 | 0.88, 1.09 | 0.687 |
| Ca_max (time dependent) | 1.00 | 0.98, 1.02 | 0.846 |
| Na_max | 1.02 | 1.00, 1.03 | 0.004 |
| K_max (time dependent) | 1.02 | 1.00, 1.04 | 0.136 |
| ^1^HR = Hazard Ratio, CI = Confidence Interval | | | |

Supplementary Table 24. Survey-weighted Cox model adjusted with Boruta selected covariates (Variables other than the group listed in the table) using IPTW for 1-year mortality of cohort

| **Characteristic** | **HR^1^** | **95% CI^1^** | **p-value** |
| --- | --- | --- | --- |
| Group (time dependent) |  |  |  |
| non-SIMI | 1.00 | Reference |  |
| SIMI | 1.03 | 0.99, 1.08 | 0.169 |
| K_max (time dependent) | 1.01 | 0.99, 1.04 | 0.175 |
| K_min (time dependent) | 1.00 | 0.95, 1.05 | 0.906 |
| Na_max (time dependent) | 1.00 | 1.00, 1.00 | 0.11 |
| Na_min (time dependent) | 1.00 | 1.00, 1.00 | 0.847 |
| Ca_max (time dependent) | 1.00 | 0.98, 1.02 | 0.997 |
| ABL_max | 0.95 | 0.79, 1.14 | 0.568 |
| ABL_min (time dependent) | 0.97 | 0.93, 1.02 | 0.302 |
| AST_max | 1.00 | 1.00, 1.00 | <0.001 |
| ALT_max | 1.00 | 1.00, 1.00 | 0.006 |
| PT_max (time dependent) | 1.00 | 1.00, 1.00 | 0.904 |
| PT_min (time dependent) | 1.00 | 0.99, 1.01 | 0.933 |
| FIB_max | 1.00 | 1.00, 1.00 | 0.27 |
| FIB_min (time dependent) | 1.00 | 1.00, 1.00 | 0.869 |
| DDimer_max (time dependent) | 1.00 | 1.00, 1.00 | 0.438 |
| DDimer_min | 1.00 | 1.00, 1.00 | 0.276 |
| INR_max (time dependent) | 1.01 | 0.98, 1.04 | 0.445 |
| INR_min (time dependent) | 1.06 | 0.94, 1.19 | 0.333 |
| PLT_max (time dependent) | 1.00 | 1.00, 1.00 | <0.001 |
| PLT_min (time dependent) | 1.00 | 1.00, 1.00 | 0.324 |
| HB_max (time dependent) | 1.01 | 1.00, 1.03 | 0.049 |
| HB_min (time dependent) | 0.99 | 0.97, 1.01 | 0.159 |
| WBC_max (time dependent) | 1.00 | 1.00, 1.00 | 0.202 |
| WBC_min (time dependent) | 1.01 | 1.00, 1.01 | 0.012 |
| BUN_max (time dependent) | 1.00 | 1.00, 1.00 | 0.04 |
| BUN_min (time dependent) | 1.00 | 1.00, 1.00 | 0.025 |
| Creatinine_max | 0.96 | 0.90, 1.02 | 0.155 |
| BNP_max (time dependent) | 1.00 | 1.00, 1.00 | 0.127 |
| Lactate_max | 1.05 | 1.02, 1.09 | <0.001 |
| Lactate_min | 1.19 | 1.14, 1.25 | <0.001 |
| HCO3_max (time dependent) | 0.98 | 0.98, 0.99 | <0.001 |
| HCO3_min | 0.98 | 0.96, 1.01 | 0.298 |
| PCO2_max | 1.03 | 1.02, 1.04 | <0.001 |
| PCO2_min | 1.00 | 0.98, 1.01 | 0.482 |
| PO2_max | 1.00 | 1.00, 1.00 | 0.007 |
| PO2_min (time dependent) | 1.00 | 1.00, 1.00 | <0.001 |
| PH_max (time dependent) | 1.21 | 0.76, 1.94 | 0.427 |
| PH_min (time dependent) | 1.34 | 0.96, 1.86 | 0.083 |
| Temperature_max | 0.83 | 0.76, 0.92 | <0.001 |
| Temperature_min | 0.90 | 0.83, 0.97 | 0.009 |
| Heart_Rate_max (time dependent) | 1.00 | 1.00, 1.00 | 0.191 |
| MAP_min (time dependent) | 1.00 | 1.00, 1.00 | 0.008 |
| Vasopressor (time dependent) |  |  |  |
| YES | 1.00 | Reference |  |
| NO | 0.96 | 0.91, 1.01 | 0.145 |
| Continuous_Renal_Replacement_Therapy |  |  |  |
| YES | 1.00 | Reference |  |
| NO | 0.87 | 0.63, 1.22 | 0.427 |
| Mechanical_ventilation (time dependent) |  |  |  |
| YES | 1.00 | Reference |  |
| NO | 0.94 | 0.89, 1.00 | 0.039 |
| Age (time dependent) | 1.00 | 1.00, 1.01 | <0.001 |
| ^1^HR = Hazard Ratio, CI = Confidence Interval | | | |
